# Enhancing greenhouse cucumber production and quality utilizing organic residues as potting media through various cultivation modes and assessing application economics

**DOI:** 10.1038/s41598-025-05242-3

**Published:** 2025-06-20

**Authors:** Eman A. Gab Allah, Mohamed M. Shahein, Mohamed E. Abuarab, Victor Shaker, Emad A. Abdeldaym

**Affiliations:** 1 Department of Vegetable, Faculty of Agriculture, Cairo University, Giza, 12613 Egypt; 2 Agricultural Engineering Department, Faculty of Agriculture, Cairo University, Giza, 12613 Egypt; 3 Department of Agricultural Economics, Faculty of Agriculture, Cairo University, Giza, 12613 Egypt

**Keywords:** Substrate media culture, Cucumber, Growth, Irrigation water productivity, Fruit quality, Photosynthesis, Hydrology, Natural hazards, Climate-change impacts, Agroecology, Plant development

## Abstract

Cocopeat is among the most frequently utilized substrates in soilless farming. Nonetheless, the extraction of Cocopeat generates a detrimental carbon footprint, highlighting the necessity for alternative, sustainable substrate options. To tackle this issue, we examined the effects of substituting Cocopeat with a blend of various Rice straw, Sawdust, and compost on cucumber growth and yield over two growing seasons, 2021–2022 and 2022–2023. The treatments included Cocopeat 100% (control), sawdust 100%, rice straw 100%, compost 100%, combinations of Cocopeat and sawdust (1:1, v/v), combinations of Cocopeat and sawdust (3:1, v/v), combinations of Cocopeat and rice straw (1:1, v/v), combinations of Cocopeat and rice straw (3:1, v/v), combinations of Cocopeat and compost (1:1, v/v), and combinations of Cocopeat and compost (3:1, v/v). The highest yield was recorded with rice straw at 100.55 ton ha^− 1^, followed by the Coco 50%: Compost 50% treatment yielding 74.32 ton ha-1 and 69.26 ton ha^− 1^, respectively, while the lowest yield was noted for sawdust at 22.23 ton ha^− 1^. Across both growth seasons, rice straw achieved the highest irrigation water productivity (IWP) of 51.56 and 51.91 kg m^− 3^, respectively, followed by Coco 50%: Rice straw 50% at 38.08 and 38.37 kg m^− 3^, whereas sawdust resulted in the lowest IWPs of 6.93 and 11.48 kg m^− 3^. In both growing seasons, the rice straw showed the greatest rate of photosynthesis, with readings of 23.34 µmol m^–2^ s^–1^ and 22.14 µmol m^–2^ s^–1^, respectively. Conversely, the lowest photosynthesis rates during both growing seasons were observed with the Coco 75%: Compost 25% treatment, at 3.23 µmol m^–2^ s^–1^ and 3.03 µmol m^–2^ s^–1^, respectively. The treated rice straw substrate media ranked as the most profitable and resilient option in terms of net present value (NPV) and benefit-cost (B/C) ratio metrics, followed closely by the compost treatment. It seems that treated rice straw-based media is a promising substrate in soilless culture systems as a viable alternative substrate for cucumber cultivation instead of Cocopeat substrate.

## Introduction

Cucumber (*Cucumis Sativus*) is a major vegetable crop extensively cultivated in both open fields and controlled growing environments like greenhouses, belonging to the *Cucurbitaceae* family. In global agricultural production, cucumber follows potato, tomato, and onion in ranking. The cultivation of cucumbers within greenhouse settings is considered a highly lucrative cash crop, with a consistent increase in its cultivated area worldwide^1^. The trend of greenhouse cucumber farming has become a vital agricultural approach globally, spurred by the rising demand for vegetables and the limitations of available farmland^2^. Continuous intensive cultivation practices within greenhouse environments may result in environmental issues, such as disturbances in soil microbial diversity, an increase in soil-borne pathogens, and soil salinization, which could negatively impact cucumber yields in these settings^3^. As a result, cultivating cucumbers in protective environments requires management strategies that emphasize environmental sustainability while achieving a balance between productivity and economic feasibility^1,4,5^.

The main challenges faced in greenhouse cucumber farming may include soil salinization and degradation, nitrate pollution in groundwater, and thermal stress^6^. These concerns have become major obstacles to cucumber cultivation in greenhouse systems, especially given the changing climate marked by higher CO_2_ concentrations, rising temperatures, and modified precipitation patterns^7^. Cocopeat has proven effective as a soil amendment in ornamental nurseries for various species. However, both peat moss and Cocopeat are expensive growth media. The rising costs associated with peat moss and Cocopeat, resulting from the gradual depletion of natural reserves, have encouraged researchers to explore new soilless alternatives that are cost-efficient, productive, recyclable, suitable, biodegradable, sustainable, and environmentally friendly. Soilless cultivation could be more economical and may produce greater yields compared to traditional soil-based approaches^8^.

The intersection of environmental and economic challenges, along with the global depletion of natural resources, has spurred heightened research within the bio-economy framework, where recycling waste materials and by-products plays a crucial role. A variety of organic wastes are being incorporated into soilless cultivation practices^9^. These organic materials offer an excellent physicochemical profile, containing vital components like carbon, nitrogen, carbohydrates, and proteins essential for plant growth and development^10^. Typically, plants are grown on soilless substrates to boost profitability, operational efficiency, handling, and distribution capabilities. With lower initial pathogen infestation rates and superior physical and chemical properties such as effective root penetration and improved water permeability, commercially grown greenhouse crops like onion, pepper, tomato, cucumber, garlic, and chilies may achieve higher productivity when cultivated in soilless substrates^11^. The soilless cultivation model promotes a controlled environment that supports plant growth and development by ensuring a sufficient supply of water and nutrients. Nevertheless, the widespread use of peat as a soilless substrate in commercial nurseries has led to the depletion of peatland ecosystems, significantly diminishing irreplaceable resources^12^. Recent peat extraction efforts have caused the destruction and degradation of fragile wetland habitats worldwide, raising considerable environmental and ecological concerns regarding its use as a growth medium. The depletion of peatlands, combined with an increasing demand for peat, has led to rising peat prices^12^.

Composting, which is a regulated biological process, aids in converting decomposable organic materials into stable end-products through the involvement of microorganisms. Furthermore, soil invertebrates such as earthworms, termites, and ants play a significant role in this transformation by decomposing larger organic matter^13^. The benefits of composting include improvements in soil quality and agricultural productivity, carbon sequestration as soil organic matter, and a decrease in greenhouse gas emissions from agricultural practices^14^. Furthermore, compost has been employed to reduce soil toxicity and decrease the mobility of metals by promoting the stabilization of these metals through processes such as precipitation, complexation, and sorption^15^. Additionally, compost can function as an exceptionally effective side-dress fertilizer, enhancing the growth of lettuce plants by up to 6.8 times when combined with soil amendments^16^.

Cocopeat, an agricultural byproduct obtained from coconut husks through fiber extraction, is yet another effective amendment. The addition of Cocopeat as a bulking agent alongside other composting materials offers numerous benefits^17^. For instance, Cocopeat enhances the porosity of substrates, which improves aeration, while its high water retention capacity helps maintain optimal moisture levels^18^.

Rice is among the most widely cultivated food crops, serving as a staple for nearly half of the global population, with total production reaching 756.74 million tons. Remarkably, merely 20% of the rice straw produced globally is utilized, leading to over 100 million tons being burned each year^19^. The practice of open-field burning of rice straw significantly contributes to local air pollution and poses health hazards^20,21^. Managing rice straw, particularly the common practice of burning residues, presents complicated challenges with significant environmental and public health implications. A large volume of straw remains after harvest, often disposed of through burning, which releases various pollutants into the atmosphere. Carbon dioxide (CO_2_) accounts for 70% of these emissions, with methane (CH_4_) at 0.66%, carbon monoxide (CO) at 7%, and nitrous oxide (N_2_O) at 2.09%. This incineration process further worsens the depletion of vital soil nutrients like nitrogen and organic matter^22^. Therefore, it is crucial to explore economically feasible conversion technologies for rice straw to improve field return strategies. Sawdust may provide a sustainable alternative to peat as a growth medium^23^. Sawdust offers beneficial characteristics such as excellent drainage, increased surface area per volume, lower specific gravity, and enhanced physical features, including water retention and porosity^24^.

Due to the importance of rice as a food in many countries, the amount of waste resulting from it is considered a danger to the environment, as only 20% of it is used, and the rest is burned, which harms the environment due to emissions, in addition to the lack of economic benefits of this process. Hence, the idea of ​​​​the research came about: Why is rice straw not used as a growing medium for vegetable crops. In addition to its cheap price, large availability, and lack of benefit from it, it is considered a good growing medium due to its high porosity and containing many elements useful to the plant. Hence, the idea of ​​​​the research into the use of rice straw and compare it with the applied growing medium, which is Cocopeat, technically and financially, while making different mixtures of growing media and evaluating them in terms of productivity and quality characteristics, in addition to the economic feasibility of its application. This is because, despite the spread of the growing medium Cocopeat, its material cost is high, especially in developing countries that represent the largest producers of crops, such as Egypt, which hinders its spread and increases the costs of producing vegetables in greenhouses. Many previous studies have applied compost or Cocopeat as a growing medium due to their numerous environmental benefits, improving soil properties and increasing productivity. However, few have made mixtures of rice straw and these media to determine the combined effect they have on the productivity and quality characteristics of vegetable crops, in addition to the economic evaluation of these media, which is the most important part of the extent to which farmers are convinced of the application of these media and the extent of their spread.

This study was conducted to assess: (i) the effects of various substrate culture media (including rice straw, sawdust waste, compost, and Cocopeat) on specific morphological, physiological, and biochemical characteristics of cucumbers; (ii) to analyze the effects of different growth media on crop yield and water use efficiency, while also considering economic factors, as producers aim to boost profitability by minimizing infrastructure costs and maximizing crop output; and (iii) to investigate the ideal cultivation conditions for substrate culture media under water-scarce situations, to enhance both the quantity and quality of cucumber yields as well as water productivity in a controlled plastic greenhouse setting.

## Materials and methods

### Materials

The Vitazad Company, based in Cairo, Egypt, provided the F1 hybrid cucumber seeds. Additionally, the hybrid seeds used in this study comply with Egyptian legal requirements. Bright F1 was the cucumber hybrid utilized in this study. The substrates, which consisted of sand and compost that was derived from vegetative green waste, along with their various combinations, underwent a rigorous composting process that lasted for three months.

### Study area and growth conditions

Using cucumber plants (*Cucumis sativus* L.) of the Hybrid Bright variety, the field experiment was carried out at the Mohamed Naguib Agricultural Site, which is a part of the National Protected Cultivation in EL-Alameen, Egypt (latitude 30.8225 N, longitude 28.9543 E, with an average elevation of 20.93 m above sea level). It took place from October 20 to February 21 of two consecutive winter seasons, 2021–2022 and 2022–2023.

Because the soil is calcareous and contains a lot of calcium carbonate, Cocopeat molds were used in place of soil in the experimental setup. Despite providing fewer nutrients to plants than traditional garden soil, Cocopeat’s qualities as a soilless growth medium offer better aeration, improved drainage, and superior moisture retention, all of which are essential for plant growth. 20 cm in width, 100 cm in length, and 30 cm in thickness were the measurements of each mold. The substitute substrate (Cocopeat) and the irrigaton water were subjected to a chemical analysis (Tables 1 and 2).

**Table 1:** Chemical composition of Cocopeat molds.

pH	EC_e_ (dS m^− 1^)	*N* ^+^ (mg kg^− 1^)	*P* (mg kg^− 1^)	K^+^ (mg kg^− 1^)	Fe^2+^ (mg kg^− 1^)	Cu^2+^ (mg kg^− 1^)	Mn^2+^ (mg kg^− 1^)	Zn^2+^ (mg kg^− 1^)
7.85	0.83	90.0	7.44	362.0	0.878	0.052	0.370	0.10

ND: Not detected.

**Table 2:** Irrigation water chemical analysis at the experimental location.

pH	EC (dS m^− 1^)	TDS (ppm)	Soluble cations (mg L^− 1^)				Soluble anions (mg L^− 1^)				SAR (%)
			Ca^2+^	Mg^2+^	Na^+^	K^+^	$\:{\mathbf{C}\mathbf{O}}_{3}^{2-}$	$\:{\mathbf{H}\mathbf{C}\mathbf{O}}_{3}^{-}$	Cl^−^	$\:{\mathbf{S}\mathbf{O}}_{4}^{2-}$	
7.89	0.8	512.0	71.4	39.61	83.91	8.21	ND	226.38	131.52	163.78	1.99

### Environmental conditions

The experimental process was executed within a carefully controlled greenhouse, where the climatic conditions were characterized as arid, featuring a cold winter and a humid summer. Throughout both growing seasons, average environmental variables such as air temperature inside and outside the greenhouse, relative humidity levels, and solar radiation were documented daily from October to February (Table 3).

**Table 3:** Monthly environmental condition variables in the greenhouse as an average for the two cultivated seasons.

Growing Season	Climate parameter	October	November	December	January	February
2021–2022	T_in_ (°C)	21.93	19.98	13.71	12.29	13.36
	T_out_ (°C)	20.58	18.90	12.31	10.54	11.43
	RH_in_ (%)	73.32	77.04	77.12	73.66	74.62
	RH_out_ (%)	78.39	80.19	79.75	73.10	75.98
	Solar radiation (W m^− 2^)	369.50	328.10	274.74	299.13	339.76
2022–2023	T_in_ (°C)	22.37	20.38	13.98	12.54	13.63
	T_out_ (°C)	20.99	19.28	12.56	10.75	11.66
	RH_in_ (%)	74.79	78.58	78.66	75.13	76.11
	RH_out_ (%)	79.96	81.79	81.35	74.56	77.50
	Solar radiation (W m^− 2^)	376.89	334.66	280.23	305.11	346.56

### Irrigation water requirements

The calculation of crop evapotranspiration adhered to the guidelines set forth by the Food and Agriculture Organization of the United Nations, FAO. The reference evapotranspiration (ETo) was calculated using the Modified Penman-Monteith equation, FAO 56^25^, applying daily meteorological data collected from the experimental site. ETo calculations were facilitated using the FAO calculator (http://www.fao.org/land-water/databases-and-software/eto-calculator/en/). Additionally, the CLIMWAT 2.0 and CROPWAT 8.0 software programs were employed in accordance with Eq. 1 to estimate the evapotranspiration for cucumber.1

where ETo represents the reference evapotranspiration (mm day^− 1^), Rn indicates the net radiation at the crop surface (MJ m^− 2^ day^− 1^), G denotes the soil heat flux density (MJ m^− 2^ day^− 1^), T refers to the average daily air temperature at a height of 2 m (°C), U_2_ is the wind speed at 2 m height (m s^− 1^), and e_s_ and e_a_ signifies the saturated and actual vapor pressure deficit (kPa). The estimation of the crop’s evapotranspiration is based on the methods and procedures outlined in FAO Irrigation and Drainage Paper No. 56^25^, which details the daily water uptake of the plant through the combination of water evaporation from the soil and transpiration from the plant’s leaf stomata:

2 $$\:{\text{E}\text{T}}_{\text{C}}={ET}_{O}\:\times\:Kc\:$$

where Kc represents the crop coefficient, and ETo refers to the daily reference evapotranspiration (mm day^− 1^). The gross irrigation needs for green beans were calculated using the formula suggested by Brouwer and Heibloem^26^:

3 $$\:{IR}_{g}=\left(\frac{{ET}_{O}\times\:{K}_{C}\times\:{K}_{r}}{{E}_{i}}\right)-(R+LR)$$

where IRg denotes the gross irrigation requirements measured in millimeters per day (mm day-1), ETo signifies the reference evapotranspiration also expressed in millimeters per day (mm day-1), Kc represents the crop coefficient as documented by Allen^27^, Kr illustrates the ground cover reduction factor, and the specific values attributed to Kr shall be evaluated by Keller’s Eq. (3) as referenced by Sefrin, Riese^28^, detailed as follows:

4 $$\:{K}_{r}=GC+0.15(1-GC)$$

where GC refers to the ground cover percentage (%), which is determined by calculating the ratio of the shaded area attributable to each plant in comparison to the total area occupied by the plant, Ei indicates the irrigation efficiency expressed as a percentage (%), R represents the volume of water that the plant can acquire from sources other than the irrigation process, measured in millimeters (mm), and LR denotes the volume of water that is necessary for the effective leaching of salts, also quantified in millimeters (mm).

A comparative analysis was meticulously undertaken between the calculated irrigation water requirements, which were derived using the Penman-Monteith equation, and the actual water needs of the plant, facilitated by the installation of subsurface drainage pipes strategically placed beneath the Cocopeat molds at various designated locations throughout the experimental area, aimed at quantifying the amounts of drainage water produced, thereby permitting adjustments to the volume of applied irrigation water in a manner that conserves water resources and fosters sustainable practices in water management.

### System installation and experimental treatments

The experimental study was carried out within a climate-controlled greenhouse, the structure of which is enveloped in polyethylene and covers an expansive area of 10,500 square meters, characterized by dimensions of 100 m in width, 105 m in length, and 9 m in height. The implementation of the experimental treatments was executed through the application of a randomized complete block design (RCBD) methodology, with each treatment being replicated three times to ensure validity and reliability of results. A total of ten distinct treatments involving various growth media were employed, with Cocopeat designated as the control treatment, alongside three other media comprising sawdust, rice straw, and compost, as well as combinations of these media according to previous studies of Čepulienė, Butkevičienė^29–32^ at specific ratios of 1:1 (v/v) and 1:3 (v/v) for all conceivable pairwise mixtures, specifically including; Cocopeat 100% as the control, sawdust 100%, rice straw 100%, compost 100%, mixtures of Cocopeat and sawdust in a 1:1 ratio (v/v), mixtures of Cocopeat and sawdust in a 3:1 ratio (v/v), mixtures of Cocopeat and rice straw in a 1:1 ratio (v/v), mixtures of Cocopeat and rice straw in a 3:1 ratio (v/v), mixtures of Cocopeat and compost in a 1:1 ratio (v/v), and mixtures of Cocopeat and compost in a 3:1 ratio (v/v). A comprehensive summary of the chemical properties associated with the various media that were examined is presented in Table 4. The chemical composition of the rice straw employed in the field trials was cellulose (32%), nitrogen-free extract (1.90%), potassium (0.21%), lignin (13.3%), calcium (0.16%), phosphor (0.49%), magnesium (0.12%), sulfur (0.07%), copper (0.51 mg kg^− 1^), manganese (0.42 mg kg^− 1^), ash (18.30%), and silica (12.5%).

**Table 4:** Some chemical properties of the studied media after composting.

Growth media	Organic matter (%)	Organic carbon	Moisture content (%)	EC (dS m^− 1^)	pH	*N* (%)	*P* (%)	C/*N* ratio
Cocopeat (control)	65.2	34.3	38.3	6.2	6.8	1.5	0.17	22.9
Rice straw	52.2	27.5	40.0	7.8	7.3	1.9	0.49	14.5
Sawdust	85.8	45.1	36.7	6.1	7.3	1.2	0.1	23.7
Compost	15.83	9.18	9.0	1.38	8.66	0.63	1.28	15.0
Coco 50%: Rice straw 50%	71.72	39.84	25.65	4.56	7.51	1.44	0.13	28.0
Coco 75%: Rice straw 25%	68.46	37.07	31.98	5.38	7.15	1.47	0.15	25.0
Coco 50%: Sawdust 50%	81.77	45.67	23.15	3.43	7.44	1.05	0.10	44.0
Coco 75%: Sawdust 25%	73.49	39.99	30.73	4.82	7.12	1.27	0.13	31.0
Coco 50% : Compost 50%	40.52	21.74	23.65	3.79	7.73	1.15	0.73	20.0
Coco 75% : Compost 25%	52.86	28.02	30.98	5.00	7.27	1.28	0.45	22.0

The substrates, which consisted of sand and compost that was derived from vegetative green waste, along with their various combinations, underwent a rigorous composting process that lasted for three months, with the primary objective being to achieve a carbon-to-nitrogen (C/N) ratio of 1:20. To facilitate this process, the substrates were thoroughly mixed with prescribed quantities of poultry manure, and the composting procedure was meticulously executed within aerated mounds. These mounds were subjected to mechanical aeration that was closely monitored and adjusted with thermal fluctuations, while water was judiciously introduced to maintain the moisture level at an optimal range of approximately 60%.

Cucumber seeds were meticulously sown in propagation trays on the date of September 20, and following 30 days, the seedlings were subsequently transplanted into their designated growing media. The plants were strategically positioned within white plastic bags that had a capacity of 57 L, with dimensions measuring 75 by 25 centimeters, which were appropriately sized to accommodate two cucumber plants per bag. The spacing between individual plants within the same row was meticulously maintained at a distance of 50 centimeters, thereby ensuring optimal growth conditions.

The implementation of a surface drip irrigation system was meticulously commenced in September in the year 2021, taking place within a highly controlled environment, which notably included a sophisticated screening filter that possessed backflush capabilities along with an advanced.

fertilizer injection apparatus, specifically designed as a Venturi meter. In this carefully orchestrated setup, a drip tape known as Euro drip GR was precisely installed along the ridges of the cultivation area, with each plot being equipped with a singular lateral line to ensure optimal water distribution. The drip irrigation system that was established was further enhanced with drippers that were strategically spaced at intervals of 40 centimeters, with each dripper being engineered to deliver a consistent application rate of 2.4 L per hour, thereby ensuring efficient water usage and crop hydration.

In preparation for the planting phase, the land was thoroughly cultivated and meticulously prepared utilizing Cocopeat molds, which served as an essential component of the planting process, and a layer of sand mulch was judiciously applied to enhance soil moisture retention and regulate temperature. Following the preparation of the land, seeds were carefully sown in germination trays.

after September, ensuring that they received the optimal conditions for initial growth, and these trays were subsequently transported for planting within the greenhouse environment during the middle of October, thereby facilitating a seamless transition from germination to the growing phase.

### Measurements

#### Irrigation water productivity (IWP)

Water productivity (WP) serves as a metric for evaluating the efficient utilization of irrigation water in agricultural production. WP was calculated as delineated in the prior literature^33^ as follows:

5 $$\:IWP=\frac{{E}_{y}}{{\text{I}}_{r}}$$

where WP symbolizes the water productivity associated with the particular crop (kg m⁻³), E_y_ signifies the economic yield expressed (kg ha⁻¹), and I_r_ represents the volume of irrigation water that has been applied (m³ ha⁻¹).

#### Growth measurements

In ninety days following the transplantation process, a systematic selection of plant samples was conducted at random intervals to meticulously document various growth parameters that are critical for assessing the plants’ development. These growth parameters encompassed an array of metrics, including but not limited to the height of the plants, the count of leaves present, the surface area of the leaves, as well as both the fresh and dry weights of the plants, the number of shoots produced per individual plant, and the leaf greenness index, known as the SPAD value. A measuring tape was carefully used to estimate the plant height and root length, and a digital balance was used to exactly weigh the samples that were taken, both fresh and dry. A SPAD meter, specifically the SPAD 502 model manufactured by Minolta Co., based in Osaka, Japan, was used to carefully measure the leaf greenness index. Four separate SPAD measurements were taken around the fifth leaf of the cucumber plants, and the average value obtained from these readings was then calculated to ensure accuracy.

#### Photosynthesis measurements

To determine the intercellular carbon dioxide (CO₂) concentration, net photosynthetic rate, leaf stomatal conductance, and water use efficiency over a four-week period, six plants were randomly chosen from each treatment. Five leaves per plant, particularly the fifth leaf from the top, were used to measure photosynthetic characteristics using an infrared gas analyzer (IRGA) called LICOR 6400 (Lincoln, NE, USA). The sampling chamber’s light intensity (photosynthetically active radiation, or PAR) was adjusted to 1500 [µmol m^− 2^s^− 1^] using a Li-6400-02 B LED light source (LI-COR). We used a LI-6400-01 CO_2_ mixer (LI-COR) to keep the CO_2_ flow into the chamber at 399 µmol. mol^− 1^ concentration.

#### Fruit quantity and quality

Two to three times a week, cucumber fruits were routinely gathered, and throughout two successive growth seasons, several metrics, such as the average weight of cucumber fruits, the total number of fruits produced per plant, and the overall yield, were carefully recorded. For precise measurement, a digital refractometer (model PR101, made by Co. Ltd. in Tokyo, Japan) was used to determine the total soluble solids in the cucumber fruit. Furthermore, the fruit’s hardness was assessed using a specialist firmness testing tool made for this application. The next sections go into detail and outline other crucial characteristics of the fruit’s quality, such as its nutrient content, carbohydrate content, and total antioxidant levels.

#### Macronutrient quantification

The macronutrient composition of the cucumber plants was meticulously measured in desiccated samples derived from both the leaves and the fruits. Fresh samples of leaves and fruits underwent a thorough drying process in an air-forced oven, which was set to a temperature of 70 ºC and maintained for a continuous duration of three days to ensure complete desiccation. Following this drying process, the dried samples were carefully ground into a fine powder, which was essential for ascertaining the concentrations of key macronutrients, including nitrogen (N), phosphorus (P), potassium (K), calcium (Ca), and magnesium (Mg). With minor adjustments to the usual process, the Kjeldahl method was used to calculate the total nitrogen concentration (N), as described by Jackson^34^. An acidic solution of sulfuric and Perchloric acids was mixed with about 0.5 g of either the leaf or fruit sample. The resulting mixture was heated for ten minutes at a regulated temperature of 50 oC until a clear solution formed, signifying that digestion had been successful. To precisely measure the nitrogen concentration, the mixture was allowed to cool before the total Kjeldahl nitrogen content was measured using a steam distillation procedure in combination with 80 mL of 40% sodium hydroxide and a subsequent titration with sulfuric acid (0.1 N).

According to the method outlined by Chen et al., the total phosphorus concentrations (P) in desiccated samples were measured^33^. In conclusion, 3 mL of 30% (v: v) hydrogen peroxide and 5 mL of 98% sulfuric acid were used to digest 100 mg of the dried fine powder. The digested sample was cooled to room temperature before being diluted with deionized water to a final volume of 100 mL. Using the molybdate blue method, the amount of phosphorus in the resulting solution was measured by measuring absorbance at 700 nm using a spectrophotometer.

Using the method described by Junsomboon1 and Jakmunee, the amounts of potassium (K), calcium (Ca), and magnesium (Mg) in the desiccated samples were determined^33^. In short, a solution consisting of 50 mL of water and 5 mL of HCl was used to homogenize 1 g of the ground-up material. For fifteen minutes, the homogenate was heated on a hot plate, and digestion was performed. Whatman No. 42 filter paper was used to filter the digested solution once it had cooled. Distilled water was added to the filter to bring the total volume down to 100 mL using a volumetric flask. A flame photometer device was used to measure the concentrations of the individual components.

#### Total carbohydrates quantification

Using the phosphomolybdic acid method, the total amount of carbohydrates in the leaves and fruits was measured^33^. 10 mL of 80% ethanol was used to homogenize about 2 g of the material. Following that, the mixture was filtered using Whatman filter paper (No. 1). Following the transfer of the collected residues to a 250 mL conical flask, 150 mL of distilled water and 5 mL of 95% concentrated HCl were added. After hydrolyzing the residue for half an hour, then left the solution was left to cool at ambient temperature.

Gradually, sodium carbonate (Na_2_CO_3_) was added until the extract’s pH was neutral. After being moved into a conical flask, the filtrate was concentrated for four minutes in a water bath. One milliliter of Simonyi’s reagent was then added to a glass tube containing 0.5 milliliters of the filtrate sample. After that, the obtained mixture was diluted and determined using spectrophotometry at a wavelength of 560 nm. The results regarding total carbs were presented as a percentage (%).

#### Total antioxidant activity

The Zhang et al. technique was used to determine total antioxidant activity, which is a measure of the total antioxidant components^33^. To put it briefly, 40 milliliters of a methanol solvent (Ethanol:0.1 M HCl-85:15%, v/v) was used to homogenize 4 g of the sample, and it was then sonicated for 10 min. After filtering the homogenate, the extract was gathered. 0.2 mL of the previously described extract was diluted in 3.8 mL of a methanol DPPH solution for this specific test. After being gently stirred, the obtained mixture was left at room temperature for half an hour while being kept in complete darkness. A wavelength of 517 nm was used to measure the absorbance. The following Eq. (5) was used to define the antioxidant activity as a percentage of inhibition:

6 $${\text{Antioxidant}}\,{\text{activity}}~\left( \% \right) = \frac{{A_{{517\,nm}} \,{\text{of}}\,{\text{DPPH}}\,{\text{solution}} - {\text{~}}A_{{517~nm}} \,{\text{of}}\,{\text{sample}}}}{{A_{{517~nm}} \,{\text{of}}\,{\text{DPPH}}\,{\text{solution}}}} \times 100$$

#### Free proline quantification

Using the process outlined by Bates et al., the free proline was isolated from the cucumber plants’ fruit or leaf tissues^33^. One milliliter of extraction solution containing ethanol and water (60:40 v/v) was used to homogenize about fifty milligrams of the material. The resulting homogenate was centrifuged for four minutes at 15,000 rpm after being incubated for the entire night at 5 ^o^C. Ten milliliters of distilled water were used to dilute one milliliter of the extract. After adding 5 mL of ninhydrin and 5 mL of glacial acetic acid, the solution was placed in a boiling water bath set at 100 ^o^C for 60 min. After submerging the test tubes in cold water to stop the reaction, 4 mL of toluene was used to extract the chromophore. Using spectrophotometry, the mixed supernatants were measured at 520 nm.

#### Plant hormones bioassay

IAA, GA3, and ABA were among the phytohormones that were determined using Vogel’s approach^35^. Briefly, 10 mg of freeze-dried cucumber leaves was pulverized into a fine powder. The ground samples were cleaned in a dark environment at 5 ^o^C using a solution made of 80% methanol and 2,6-bis (1,1-dimethyl ethyl)-4-methylphenol. After centrifuging the resultant extract at 4000 rpm, it was dehydrated, filtered, and evaporated at 35 ^o^C with reduced pressure. IAA, GA3, and ABA levels were measured using Ati-Unicum gas liquid chromatography. The IAA, GA3, and ABA concentrations were assessed utilizing an Ati-Unicum gas liquid chromatography 610 Series, which features a flame ionization detector. The phytohormones were separated using a coiled glass column measuring 1.5 m x 4 mm and filled with 1% OV-Gases; the flow rates for nitrogen, hydrogen, and air were set at 30, 30, and 330 mL/min, respectively. The identification and quantification of abscisic acid (ABA), Gibberellic acid (GA3), and indole acetic acid (IAA) were carried out by employing pure hormone standards and a Microsoft program to determine the concentrations of the detected peaks.

#### Growth media chemical properties

To determine each growth medium’s moisture content, the media were dried at 105 °C until their weight remained constant. The moisture content percentage was then computed. In a water extract (growth medium sample: distilled water, 1:10 by weight/volume), the electrical conductivity (EC) and pH were measured. The mixture was stirred for 15 min, left to stand for 60 min, filtered, and measurements were made using an EC meter and a pH meter, respectively. The proportion of organic matter, on the other hand, was measured after ashing at 550 °C in a furnace. Total organic carbon (TOC) was computed using Nelson and Sommers’ method in 1996, TOC = OM/1.9. According to Olsen and Sommers^36^, the phosphorus % was assessed calorimetrically using a Spectrophotometer (Models 6300 and 6100 Jenway Co.), whereas the nitrogen percentage was calculated using the approach supplied by Bremner and Mulvaney^37^.

### Statistical analysis

Before ANOVA was used, the obtained dataset was tested for homogeneity of variances (Bartlett’s test) and normality (Shapiro-Wilk test) about the residuals^38,39^. To find significant changes between treatments, the combined data from both growing seasons were subjected to an ANOVA using IBM SPSS Statistical software (version 25). This was followed by Duncan’s multiple range tests (*P* < 0.05). Pearson correlation analysis and Heatmap representation were carried out via an online statistical analysis and visualization tool^40^.

### Financial analysis

Net Present Value (NPV) and Cost-Benefit Ratio (B/C) are the two main decision criteria that are typically used to assess the economic feasibility of an investment or to rank projects. Understanding basic ideas like opportunity cost and the time worth of money is necessary for an introduction to these criteria. The fact that money obtained now is worth more than a dollar anticipated in the future is something that people naturally understand. This is because, by investing a $1 that is acquired now to generate interest, it can grow to a dollar plus interest by the designated future date. Thus, the opportunity cost is represented by the interest^41^. The discussion and equations in this part will use several different terminology and abbreviations. The following is a list of them, with the abbreviations noted for future use.

- **Present value (PV)**: The amount of money invested or available now, or the present value of a quantity or amounts to be received hereafter.
- **Future value (FV)** is the sum of money that will be received at a later period or that a present value will grow into at a later date when invested at a specific interest rate.
- **Interest rate (i)**: Also called the discount rate in some applications, it is the interest rate used to find present and future values, often equal to the opportunity cost of capital. In the mathematical formulas presented, will be expressed as a decimal value rather than a percentage.
- **Time periods (n)**: The number of time periods to be used for computing present and future values.

7 $$\:PV=\frac{FV}{{(1+i)}^{n}}$$

#### Decision criteria

To decide which of the numerous initiatives to pursue and which to reject, the analyst must first identify, price, and analyze the costs and benefits^42^. The simplest way to calculate the project’s value using discounted cash flow is to use the NPV, which shows the present value of the additional cash flow stream or net benefit. By computing the difference between the present value of the revenue stream and the present value of the cost stream, the NPV can also be determined. This can be understood as the revenue generated by an investment, expressed in current value. If a project’s NPV is more than zero, it is deemed economically viable.

8 $$\:NPV=\sum\:_{t=1}^{n}\frac{{B}_{t}}{{(1+i)}^{t}}-\sum\:_{t=1}^{n}\frac{{C}_{t}}{{(1+i)}^{t}}$$

To determine the economic viability and efficacy of an investment, the second metric is the benefit-to-cost ratio (B/C), which serves as a financial gauge to evaluate the benefits generated per unit of cost incurred. By dividing the present value of the benefits by the present value of the expenditures, one can get the benefit-cost ratio, or B/C ratio. If a project’s B/C ratio is more than 1, it is considered economically feasible.

9 $$\:\frac{B}{C}\:Ratio=\frac{\sum\:_{t=1}^{n}\frac{{B}_{t}}{{(1+i)}^{t}}}{\sum\:_{t=1}^{n}\frac{{C}_{t}}{{(1+i)}^{t}}}$$

Importantly, a 12% discount rate is a standard in financial analysis, representing the opportunity cost in developing nations. For the sake of simplicity in the analysis, it is assumed that revenues and costs remain unchanged throughout the project’s life.

#### Sensitivity analysis (Treatment of Uncertainty)

One significant benefit of thorough financial project evaluation is its capability to analyze the impact on the project’s earning potential when actual circumstances differ from the assumptions made during planning. This emphasizes a crucial element of project analysis: forecasts are inherently subject to considerable uncertainty regarding actual outcomes^42^. Moreover, sensitivity analysis was utilized to evaluate the effect of fluctuations in key variables or assumptions on the results of a project or investment. By examining a 25% rise in costs alongside a 25% drop in revenues, sensitivity analysis assists in identifying potential risks and vulnerabilities within the investment. It enables decision-makers to understand how changes in critical parameters can influence project outcomes, cash flow, and profitability.

## Results

### Irrigation water applied and irrigation water productivity

The volume of irrigation water utilized for the various treatments of growth media remained constant because the experiment was conducted in a greenhouse under controlled conditions. Moreover, the quantity of irrigation water allocated to each treatment was standardized, enabling us to evaluate the different agricultural growth media based on their water retention capabilities. This, in turn, helps in conserving irrigation water, ensuring adequate moisture for crop growth, reducing stress on the plants, and ultimately enhancing water productivity along with cucumber yields. Accordingly, the irrigation water supplied to all treatments was maintained at 1936.73 m^3^ ha^− 1^ for both growing seasons (Table 5).

**Table 5:** Effect of different growth media on yield and water productivity.

Growth media	Irrigation Water applied (m^3^ ha^− 1^)		Yield (Mg ha^− 1^)		Irrigation water productivity (Kg m^− 3^)	
	1st	2nd	1st	2nd	1st	2nd
Cocopeat (control)	1936.73	1936.73	62.54 b	67.28 b	32.29 c	34.74 c
Rice straw	1936.73	1936.73	99.87 a	100.55 a	51.56 a	51.91 a
Sawdust	1936.73	1936.73	13.43 e	22.23 e	6.93 f	11.48 g
Compost	1936.73	1936.73	64.92 b	69.26 b	29.53 d	33.01 c
Coco 50%: Rice straw 50%	1936.73	1936.73	73.75 b	69.13 b	38.08 b	38.37 b
Coco 75%: Rice straw 25%	1936.73	1936.73	49.22 d	59.45 c	25.41 d	30.69 d
Coco 50%: Sawdust 50%	1936.73	1936.73	45.05 d	42.27 d	23.26 e	21.82 f
Coco 75%: Sawdust 25%	1936.73	1936.73	46.76 d	44.05 d	24.14 de	22.74 f
Coco 50% : Compost 50%	1936.73	1936.73	65.68 b	74.32 b	33.91 c	35.76 c
Coco 75% : Compost 25%	1936.73	1936.73	59.19 c	62.93b c	21.12 e	26.28 e

Means followed by different letters indicate significant differences between the treatments (*n* = 10; Duncan test at 95%).

In the 2021–2022 season, the rice straw treatment yielded the highest output of 99.87 tons per hectare, followed by the mixed treatments of Coco 50%: Rice straw 50% and Coco 50%: Compost 50%, which produced 73.75 Mg ha^− 1^ per hectare and 65.68 ton ha^− 1^, respectively. On the other hand, the sawdust treatment yielded the least, at 13.43 Mg ha^− 1^. The productivity metrics in the second season mirrored this trend, showing minor and negligible variations between the two growing seasons. The rice straw media culture achieved the highest yield again, at 100.55 Mg ha^− 1^, with the following yields recorded at 74.32 Mg ha^− 1^ and 69.26 Mg ha^− 1^ for the treatments of Coco 50%: Compost 50% and Compost, respectively. Conversely, the yield from sawdust remained the lowest at 22.23 Mg ha^− 1^ (Table 5).

Across both growing seasons, the rice straw growth media yielded irrigation water productivities (IWPs) of 51.56 and 51.91 kg m^− 3^, respectively, followed by Coco 50%: Rice straw 50%, with IWPs of 38.08 and 38.37 kg m^− 3^ for both seasons. In contrast, the sawdust treatment recorded the lowest IWPs of 6.93 and 11.48 kg m^− 3^ across both growing seasons. Notable differences in yield and IWP were observed among all treatments (Table 5).

### Vegetative growth parameters

**Fig. 1:**
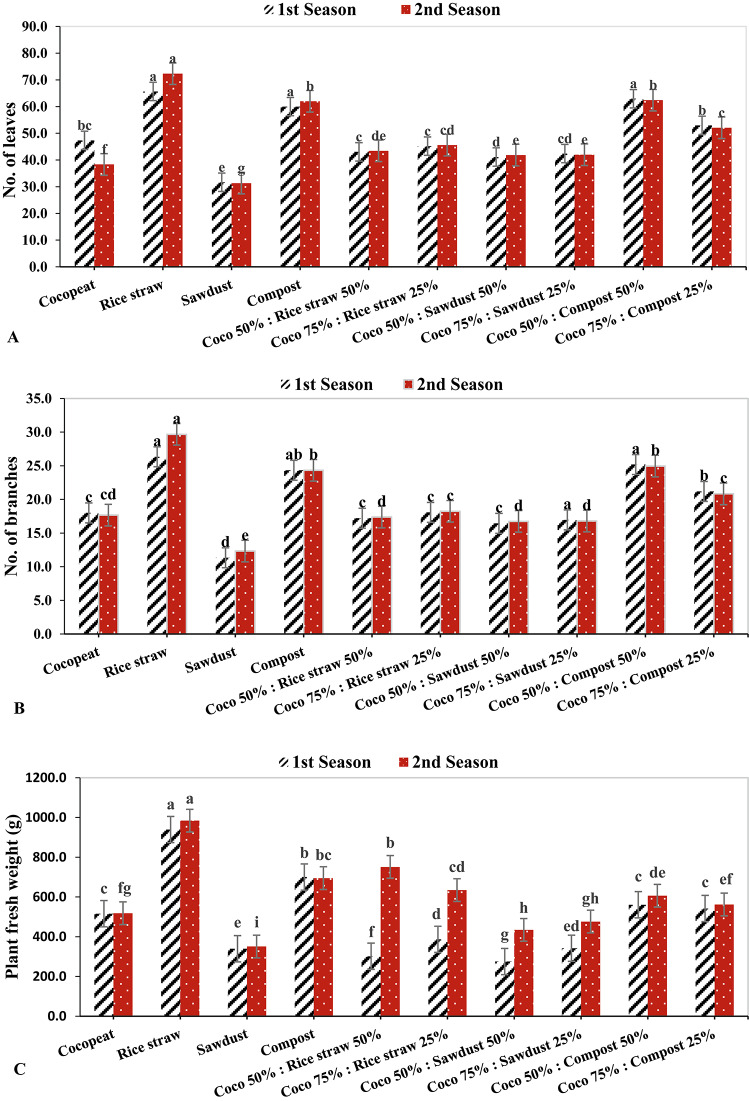

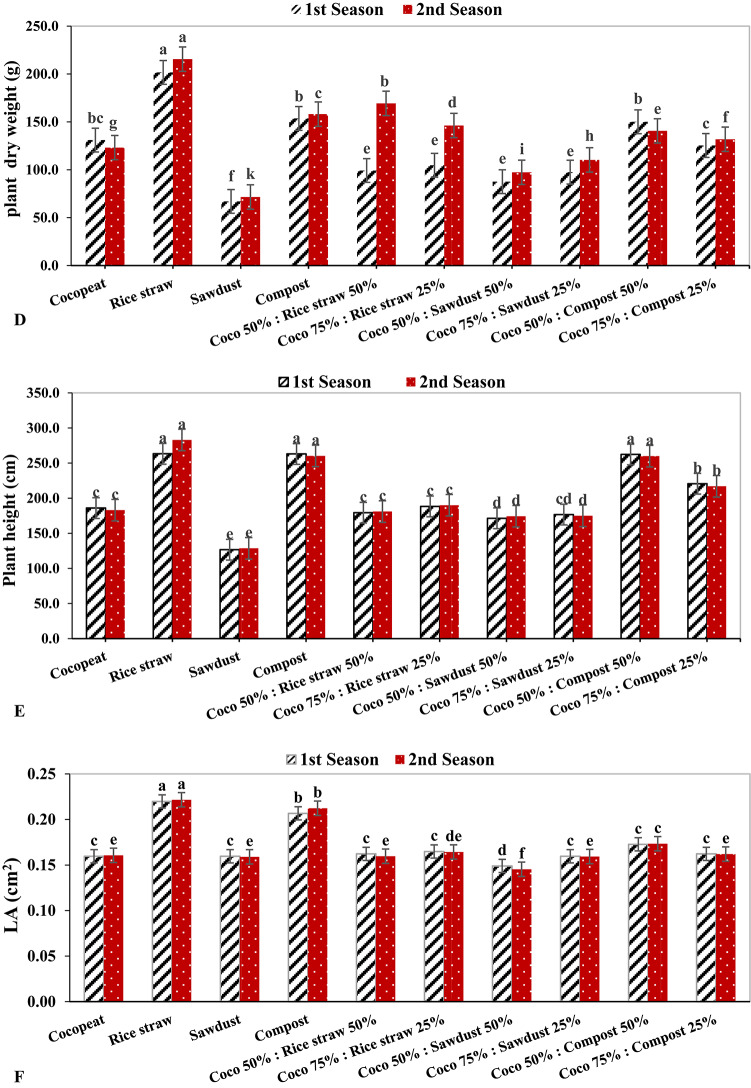

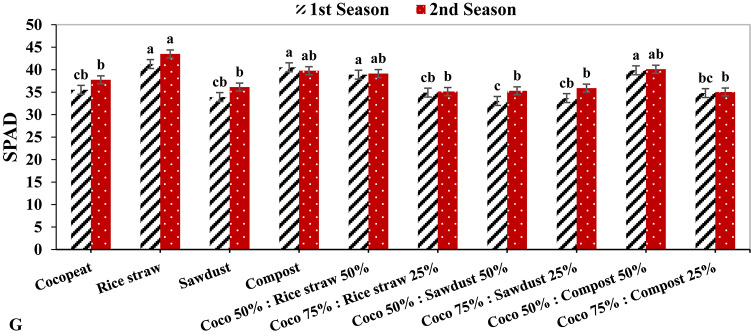
The effect of growth media on vegetative growth parameters of cucumber plants.

Cucumber plants showed significant differences between all treatments, the rice straw treatment in 2022–2023 had the highest number of leaves of 72.33, followed by treatments with Coco 50%: Compost 50% and Compost with 62.96 in 2021–2022 and 62.0 in 2022–2023, respectively. On the contrary, the sawdust treatment had the lowest number of leaves in both growing seasons with 31.67 and 31.33, respectively (Fig. 1A). On the other hand, the maximum number of branches was detected under rice straw ​​in both growing seasons with 26.33 and 29.67 respectively, followed by 50% Coco: 50% Compost with 25.18 and 24.96 in 2021–2022 and 2022–2023 respectively, conversely, the lowest number of branches was observed under sawdust growing medium with 11.33 and 12.33 in 2021–2022 and 2022–2023 respectively (Fig. 1B).

Cucumber plants grown in rice straw showed the highest fresh weight in both growing seasons 939.33 g and 984.26 g, respectively, While the plants cultivated in a sawdust treatment had the lowest fresh weight in the 2021–2022 and 2022–2023 seasons, with 339.33 g and 350.66 g, respectively (Fig. 1C). In the same context, the dry weight of the cucumber plants varies significantly among all treatments. In 2021–2022 and 2022–2023, the dry weight of cucumber plants grown on a rice straw treatment increased to 201.65 g and 215.50 g, respectively. On the other hand, during both growth seasons, the sawdust treatment decreased dry weight by 66.94 g and 71.55 g, respectively (Fig. 1D).

Plant height was significantly impacted by the variations in various growing situations, cucumber plants grown in rice straw medium reached their maximum plant heights throughout the two growing seasons by 263.33 cm and 283 cm, respectively, followed by Cocopeat 50%: Compost 50% treatment with 262.33 cm and 260 cm respectively and compost treatment with 263 cm and 260 cm respectively. In contrast, the sawdust medium produced cucumber plants with the lowest heights in both seasons with 126.67 cm and 128.67 cm, respectively (Fig. 1E). Likewise, in the both growing seasons 2021–2022 and 2022–2023, the leaf area of cucumber plants showed an increase in value when grown in rice straw, measuring 0.220 and 0.222 cm^2^, respectively. Conversely, the cucumber plants cultivated in 50% Cocopeat: 50% Sawdust had the least leaf area with 0.149 and 0.145 cm^2^ for each growing season, respectively (Fig. 1F).

The highest values of the leaf greenness index (SPAD) for cucumber were recorded under Rice straw, Compost, Coco peat 50%: Compost 50%, and Coco peat 50%: Rice straw 50% treatments in the 2021–2022 season, with respective values of 41.26, 40.53, 39.86, and 38.90. For the second growing season 2022–2023, the rice straw treatment had the highest values of the SPAD, followed by the Cocopeat 50%: Compost 50%, Compost, and the Cocopeat 50%: Rice straw 50% treatments with respective values of 43.50, 40.10, 39.77, and 39.14. Additionally, in both growing seasons, the Cocopeat 50%: Sawdust 50% treatment had the lowest values of the SPAD reading for cucumber plants with 33.06 and 35.30, respectively (Fig. 1G).

### Photosynthesis measurements

**Fig. 2:**
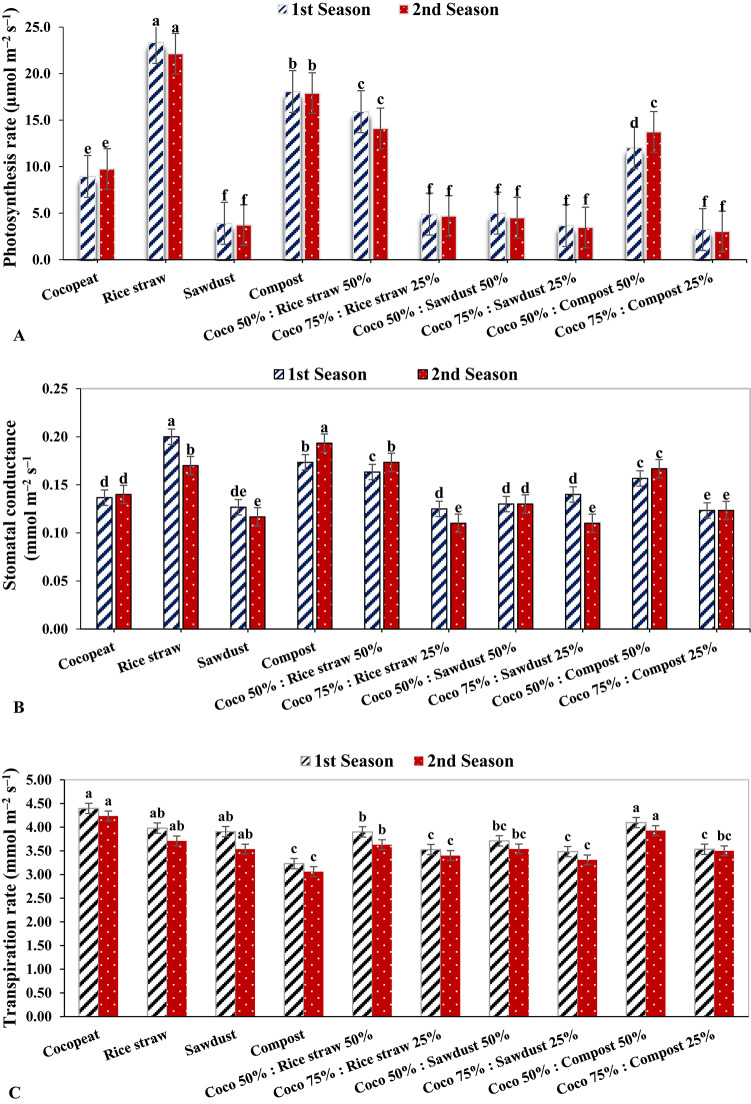

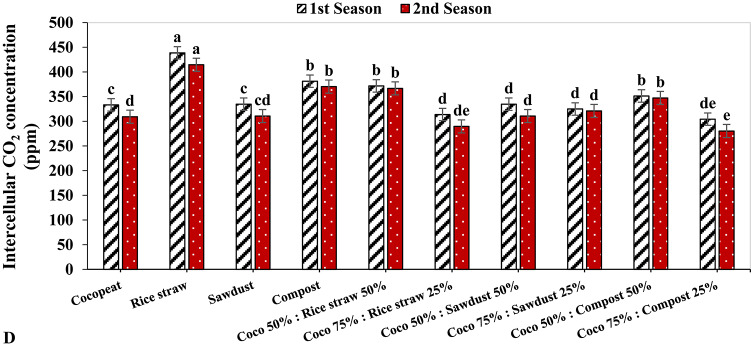
The effect of growth media on photosynthesis measurements of cucumber plants.

In both growing seasons, the rice straw treatment exhibited the highest rate of photosynthesis, with values of 23.34 µmol m^–2^ s^–1^ and 22.14 µmol m^–2^ s^–1^, respectively. In contrast, the photosynthesis rate was lowest in both growing seasons for the Coco 75%: Compost 25% treatment, by 3.23 µmol m^–2^ s^–1^ and 3.03 µmol m^–2^ s^–1^, respectively (Fig. 2A). In the same context, the greatest stomatal conductance had the highest value under rice straw growing medium during the 1st growing season by 0.20 mmol m^–2^ s^–1^, and this was followed by compost treatment during the 2nd growing season with 0.19 mmol m^–2^ s^–1^. Conversely, in the 1st growing season, the lowest stomatal conductance was detected under the Coco 75%: Sawdust 25% treatment with 0.12 mmol m^–2^ s^–1^, while in the 2nd growing season the lowest stomatal conductance was registered under Coco 75%: Compost 25% treatment (Fig. 2B).

On the other hand, the Cocopeat treatment showed the highest transpiration rate in both growth seasons, with 4.40 mmol m^–2^ s^–1^ and 4.24 mmol m^–2^ s^–1^ respectively, followed by the Coco 50%: Compost 50% treatment on both growing seasons with 4.10 mmol m^–2^ s^–1^ and 3.93 mmol m^–2^ s^–1^ respectively. On the reverse hand, the lowest transpiration rate was observed under compost treatment by 3.23 mmol m^–2^ s^–1^ and 3.06 mmol m^–2^ s^–1^, respectively (Fig. 2C). In the same context, the lowest intercellular CO_2_ concentration was obtained under the Coco 75%: Compost 25% treatment in both growing seasons at 304.33 ppm and 280.33 ppm, respectively. While the highest intercellular CO_2_ concentration was noticed under rice straw growing medium in both seasons, with 438.66 ppm and 414.66 ppm, respectively (Fig. 2D).

### Leaf quality parameters

**Fig. 3:**
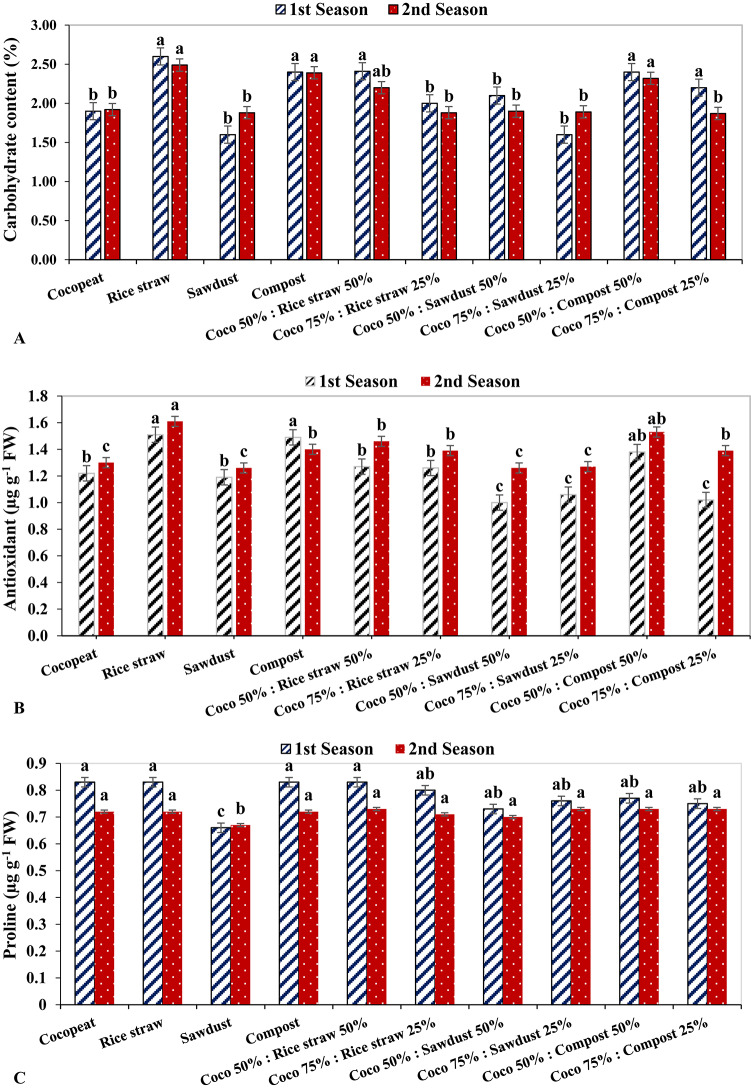
The Effect of growth media on leaf quality parameters of cucumber plants.

In 1st and 2nd growing seasons, the rice straw treatment had the highest carbohydrate content in leaf with 2.60% and 2,49% respectively. Conversely, the sawdust-growing medium had the lowest carbohydrate content in leaf by 1.60% in the 1st growing season, while the Coco 75%: Compost 25% treatment had the lowest carbohydrate content in leaf by 1.87% in the 2nd growing season (Fig. 3A). On the other hand, the Coco 50%: Compost 50% treatment had the highest level of antioxidants in leaf by 1.61 µg g^− 1^ FW, followed by the rice straw treatment with 1.53 µg g^− 1^ FW in the second growing season. In contrast, the Coco 50%: Sawdust 50% treatment had the lowest leaf antioxidant content, with 1.0 µg g^− 1^ FW, followed by the Coco 75%: Compost 25% treatment with 1.02 µg g^− 1^ FW in the first growing season (Fig. 3B).

The Proline content in cucumber leaf had the lowest levels under sawdust growing medium in both growing seasons with 0.66 µg g^− 1^ FW and 0.67 µg g^− 1^ FW, respectively, while all treatments displayed the highest level of Proline in the leaves, between 0.77 µg g^− 1^ FW and 0.83 µg g^− 1^ FW in both seasons with no significant difference between values except for sawdust treatment (Fig. 3C).

### Leaf phytohormones content

**Fig. 4:**
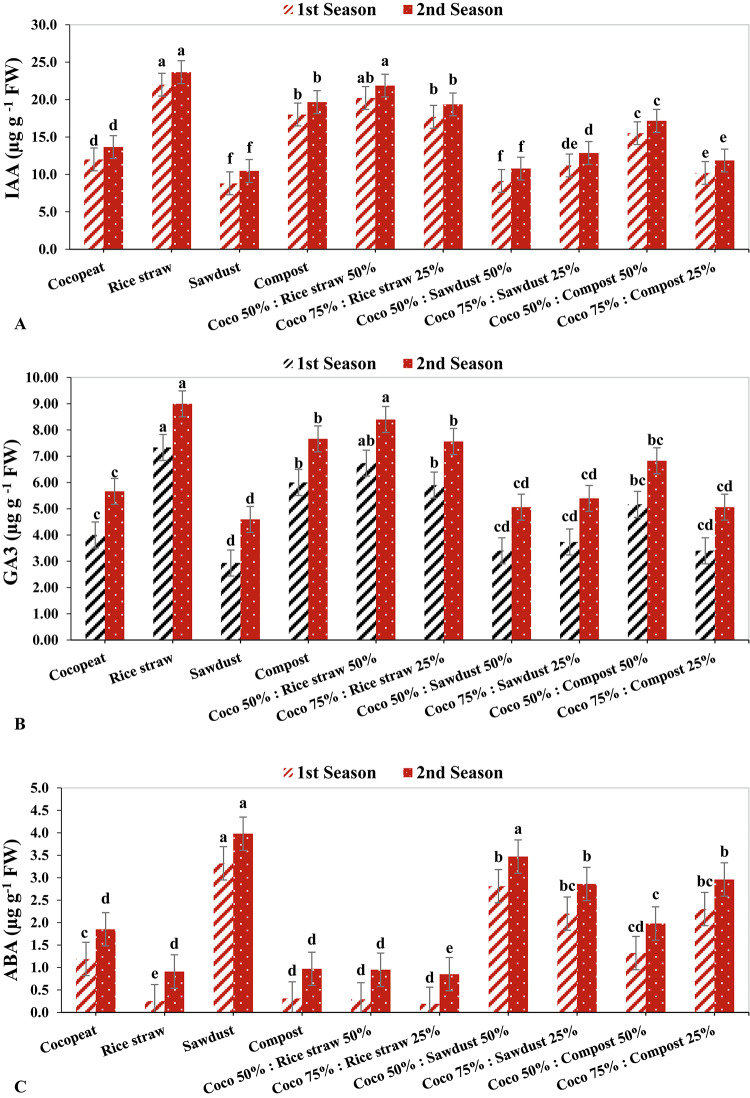
The effect of growth media on leaf concentrations of IAA (**A**), GA3 (**B**), and ABA (**C**).

The highest leaf IAA values were found in the rice straw treatment during both growing seasons (22.00 µg g^− 1^ FW and 23.66 µg g^− 1^ FW, respectively), followed by the Coco 50%: Rice straw 50% treatment in the second growing season (21.87 µg g^− 1^ FW), and the sawdust treatment during both growing seasons (8.80 µg g^− 1^ FW and 10.46 µg g^− 1^ FW, respectively) (Fig. 4A).

Additionally, in the second growing season, the maximum GA3 content was found under rice straw treatment (8.99 µg g^-1^ FW), followed by coco 50%: rice straw 50% (8.40 µg g^-1^ FW) in the same growth season, while sawdust treatment had the lowest GA3 with 2.93 µg g^-1^ FW in the 1st growing season followed by Coco 50%: Sawdust 50% with 3.40 µg g^-1^ fw in the same season (Fig. 4B). Conversely, the sawdust treatment showed highest ABA levels with 3.98 µg g^-1^ FW in the 2nd growing season followed by Coco 50%: Sawdust 50% with 3.47 µg g^-1^ FW in the same season, while the lowest levels of ABA under Coco 75%: Rice straw 25% treatment followed by rice straw treatment with 0.19 µg g^-1^ FW and 0.25 µg g^-1^ FW respectively in the 1st growing season (Fig. 4C).

### Leaf nutrient contents

The highest leaf nitrogen content (N) was observed under Coco 50%: Rice straw 50% treatment in 2nd growing season with 4.66% followed by Rice straw with 4.65%, while the lowest leaf N content was detected under Coco 75%: Sawdust 25% in 1st growing season with 3.67% followed by Coco 75%: Rice straw 25% in the same growing season with 3.79% (Table 6). On the other hand, the highest leaf phosphorus content (P) was detected under rice straw in the 2nd growing season by 1.39%, followed by Coco 50%: Rice straw 50% with 1.22% in the same growing season. Contrariwise, the lowest leaf phosphorus content was noticed under Coco 75%: Rice straw 25% treatment in the 1st growing season by 0.72% (Table 6).

**Table 6:** The effect of growth media on the leaf nutrient content of cucumber plants.

Growth media	*N* (%)		*P* (%)		K (%)		Ca (%)		Mg (%)	
	1st	2nd	1st	2nd	1st	2nd	1st	2nd	1st	2nd
Cocopeat (control)	4.39 a	3.80 b	0.91 b	0.99 b	3.32 a	3.53 b	5.35 a	5.56 b	0.84 a	0.81 a
Rice straw	4.42 a	4.65 a	1.3 a	1.39 a	3.92 a	4.04 a	6.05 a	6.66 a	0.85 a	0.93 a
Sawdust	4.51 a	3.31 b	0.89 b	0.89 b	2.52 b	2.52 c	4.85 b	4.65 cd	0.79 a	0.76 ab
Compost	4.31 a	4.44 a	1.07 a	1.05 ab	3.52 a	4.01 a	5.65 a	6.05 a	0.84 a	0.89 a
Coco 50%: Rice straw 50%	4.37 a	4.66 a	1.04 ab	1.22 a	3.62 a	3.93 ab	5.85 a	6.06 a	0.84 a	0.83 a
Coco 75%: Rice straw 25%	3.79 b	4.45 a	0.72 c	0.99 b	2.72 b	3.49 b	5.45 a	5.76 a	0.84 a	0.88 a
Coco 50%: Sawdust 50%	3.82 b	3.55 b	0.86 b	0.90 b	1.82 d	2.77 c	5.15 a	4.83 c	0.80 a	0.72 b
Coco 75%: Sawdust 25%	3.67 b	3.59 b	0.95 b	0.97 b	2.12 c	2.54 c	4.55 b	5.14 c	0.81 a	0.71 b
Coco 50%: Compost 50%	4.23 a	4.31 a	1.03 ab	1.20 a	3.52 a	3.85 ab	5.55 a	5.96 ab	0.83 a	0.84 a
Coco 75%: Compost 25%	3.82 b	3.73 b	0.86 b	0.97 b	2.42 b	3.37 b	4.52 b	5.55 b	0.82 a	0.70 b

Means followed by different letters indicate significant differences between the treatments (*n* = 10; Duncan test at 95%).

The treatment with rice straw had the highest leaf potassium content (K) in the second growing season by 4.04%, followed by compost with 4.01%. The Coco 50%: sawdust 50% treatment in the first growing season had the lowest leaf K content with 1.82%, followed by Coco 75%: Rice straw 25% in the same growing season by 2.12% (Table 6). In the same context, the lowest leaf calcium content (Ca) was observed under Coco 75%: Compost 25% treatment by 4.52% in the first growing season, followed by sawdust treatment by 4.65% in the second growing season. The highest leaf Ca content was found under rice straw in both growing seasons by 6.66% and 6.05% respectively (Table 6).

In the second growing season, the rice straw treatment had the highest leaf magnesium content (Mg) by 0.93%, followed by compost with 0.89% in the same growing season. In contrast, the lowest leaf Mg content was found under the Coco 75%: Compost 25% and Coco 75%: Sawdust 25% treatments in the second growing season by 0.70% and 0.71%, respectively (Table 6).

### Fruits quality parameters

**Fig. 5:**
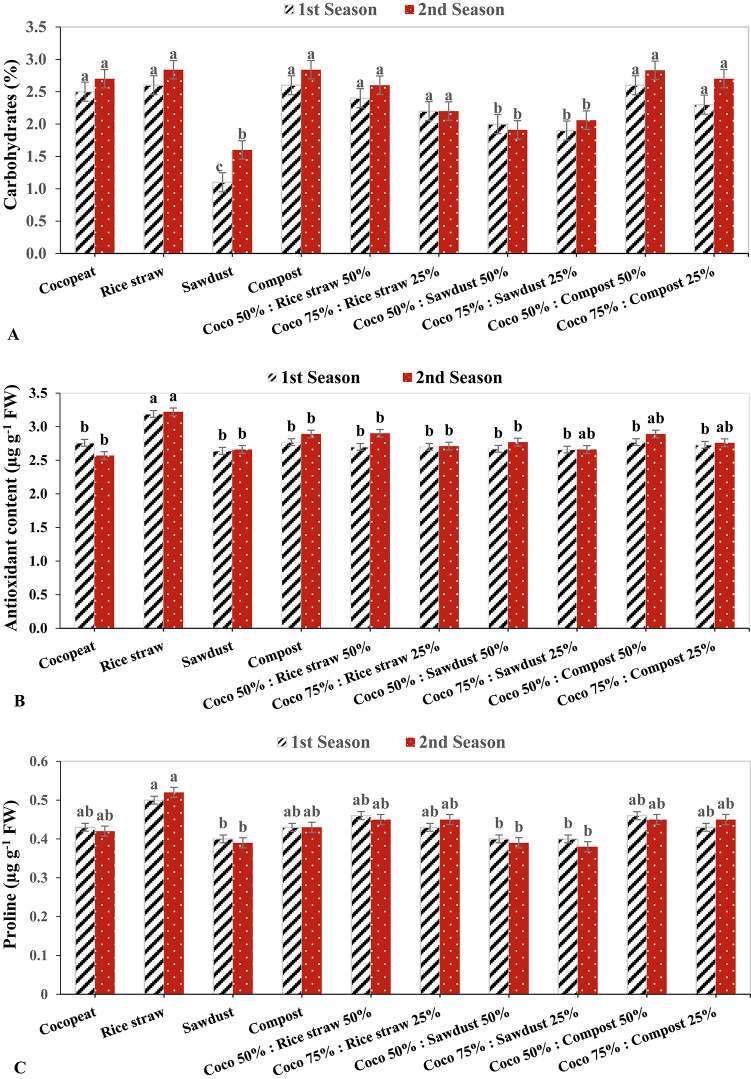

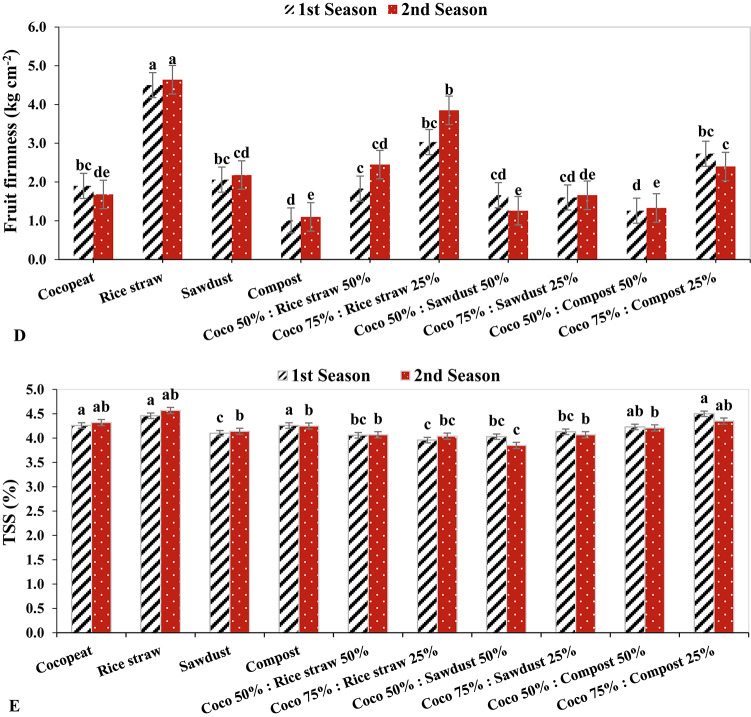
The Effect of growth media on cucumber fruit quality parameters.

In the second growing season, the highest levels of fruit carbohydrates were found under the rice straw and compost treatments at 2.84%, followed by the Coco 50%: Compost 50% treatment at 2.83%. In contrast, the lowest levels of fruit carbohydrates were found under the sawdust treatment in both growing seasons, at 1.1% and 1.6%, respectively (Fig. 5A). In the same context, in both growing seasons, rice straw had the highest antioxidant content in fruit by 3.19 µg g^−1^ FW and 3.22 µg g^-1^ FW respectively, followed by Coco 50%: Compost 50% with 2.89 µg g^−1^ FW in the 2nd growing season. On the other hand, the lowest fruit antioxidant content was observed in the 1st growing season under sawdust treatment at 2.46 µg g^-1^ FW, and in the second growing season with Cocopeat treatment with 2.57 µg g^−1^ FW (Fig. 5B).

The rice straw had the highest fruit Proline level in both growing season by 0.5 µg g^−1^ FW and 0.52 µg g^−1^ FW respectively, while in the 2nd growing season, the Coco 75%: Sawdust 25% had the lowest fruit Proline level with 0.38 µg g^-1^ FW, followed by sawdust and Coco 50%: Sawdust 50% treatment in the same growing season by 0.39 µg g^−1^ FW (Fig. 5C). In both growing seasons, cucumber plants cultivated in rice straw medium exhibited the greatest fruit firmness with 4.50 kg cm^-2^ and 4.64 kg cm^-2^, respectively, indicating a significant impact of different growing conditions on fruit firmness. Conversely, cucumber plants grown in sawdust medium had the lowest fruit firmness in both seasons at 1.01 kg cm^-2^ and 1.10 kg cm^-2^, respectively (Fig. 5D).

Similarly, in the second growing season, TSS in fruit had the highest value by 4.57% when cultivated in rice straw, followed by Coco 75%: Compost 25% treatment in the 1st growing season by 4.50%. In contrast, the Coco 50%: Sawdust 50% treatment had the lowest TSS in fruit, with 3.58% in the 2nd growing season, followed by the Coco 75%: Rice straw 25% with 3.96% in the 1st growing season (Fig. 5E).

### Fruit nutrient contents

The rice straw growing medium had the highest fruit N content in the second growing season by 3.65%, followed by compost with 3.46%. The Coco 75%: Compost 25% treatment in the first growing season had the lowest fruit N content with 2.59%, while the treatment with Coco 75%: Sawdust 25% in the second growing season had the lowest fruit N content by 2.76% (Table 7). However, under rice straw growing medium in both growing seasons, the highest fruit phosphorus level was found to be 1.19% and 1.79%, respectively. On the other hand, during both growth seasons, sawdust treatment resulted in the lowest fruit phosphorus concentration, at 0.64% and 0.62%, respectively (Table 7). During both growing seasons, the rice straw treatment exhibited the greatest fruit K content, with 3.77% and 4.26%, respectively. The first growing season’s treatment with Coco 50%: Sawdust 50% had the lowest fruit K content by 1.18%, followed by Coco 75%: Compost 25% with 1.50% in the same growing season (Table 7).

However, the lowest fruit Ca content was noted under the Coco 50%: Sawdust 50% treatment by 0.64% in the first growing season, and the Cocopeat treatment by 0.64% in the same growing season. In both growth seasons, rice straw had the greatest fruit Ca content with 0.84% and 0.92%, respectively (Table 7). The rice straw treatment had the greatest fruit Mg concentration by 0.52% in the second growing season, followed by Cocopeat with 0.51%. On the other hand, during the second growing season, the Sawdust treatment yielded the lowest fruit Mg content with 0.43%, followed by the Coco 75%: Sawdust 25% treatment in the same season by 0.45% (Table 7).

**Table 7:** The effect of growth media on the fruit nutrient content of cucumber plants.

Growth media	*N* (%)		*P* (%)		K (%)		Ca (%)		Mg (%)	
	1st	2nd	1st	2nd	1st	2nd	1st	2nd	1st	2nd
Cocopeat (control)	2.76b	3.24a	1.13a	1.31b	2.67bc	3.04b	0.64b	0.70d	0.44a	0.51a
Rice straw	3.30a	3.65a	1.19a	1.79a	3.77a	4.26a	0.84a	0.92a	0.45a	0.52a
Sawdust	3.13a	2.77b	0.64c	0.62 g	3.43ab	3.23b	0.70b	0.69de	0.45a	0.43b
Compost	3.27a	3.46a	1.05a	1.16de	3.02b	3.62a	0.75ab	0.75c	0.46a	0.46ab
Coco 50%: Rice straw 50%	3.0a	3.39a	1.09a	1.45b	2.33c	3.53ab	0.68b	0.82b	0.41a	0.47a
Coco 75%: Rice straw 25%	3.14a	3.2a	1.04a	1.30c	2.11 cd	3.33b	0.67b	0.75c	0.46a	0.48a
Coco 50%: Sawdust 50%	2.92ab	3ab	0.82b	0.95f	1.18e	3.08b	0.64b	0.69de	0.47a	0.46ab
Coco 75%: Sawdust 25%	2.81b	2.76b	0.83b	0.97f	2.06d	3.07b	0.65b	0.69e	0.47a	0.45b
Coco 50%: Compost 50%	3.05a	3.12ab	0.91b	1.06ef	2.26c	3.32b	0.66b	0.71d	0.47a	0.47a
Coco 75%: Compost 25%	2.59b	3.14ab	0.89b	1.23 cd	1.50e	3.20b	0.65b	0.7d	0.42a	0.48a

Means followed by different letters indicate significant differences between the treatments (*n* = 10; Duncan test at 95%).

### Correlation study

**Fig. 6:**
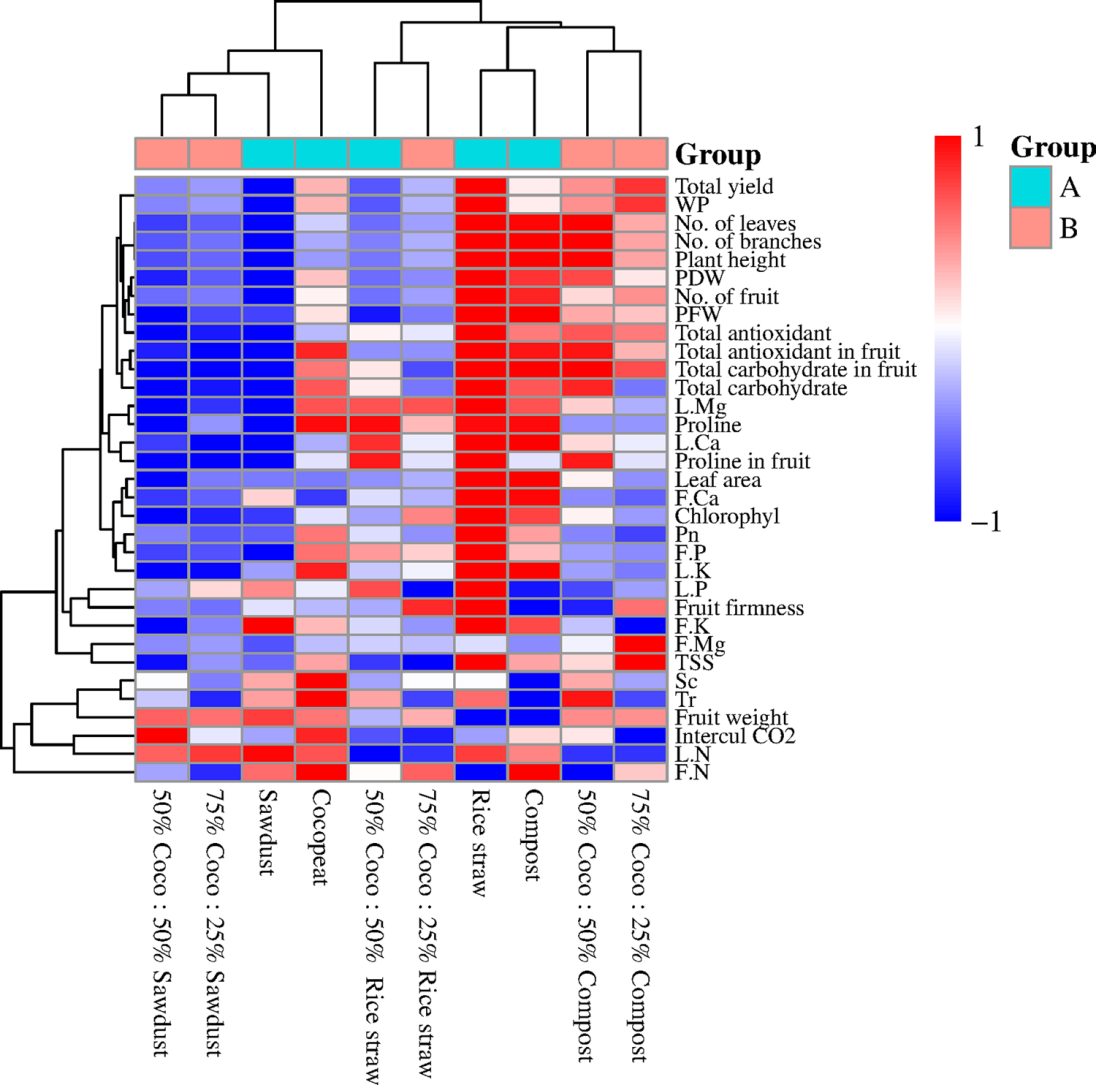
Dendrogram of two-way cluster analysis of growth media treatments.

The bi-directional cluster analysis and the resulting Dendrogram derived from various growth media treatments indicated that the Dendrogram concerning plant growth, photosynthesis, chemical composition of leaves, chemical composition of fruits, fruit quality, as well as yield and yield component parameters, where the two-way cluster analysis and the produced Dendrogram based on the 42 measurements distinctly grouped them into three categories (A, B, and C) (Fig. 6).

In line with this, each of the three categories (A, B, and C) is further divided into four and three subcategories, each consisting of one of the following: Coco peat 100% (control), rice straw 100%, sawdust 100%, compost 100%, combinations of Cocopeat and rice straw (1:1, v/v), combinations of Cocopeat and sawdust (1:1, v/v), combinations of Cocopeat and compost (1:1, v/v), combinations of Cocopeat and rice straw (3:1, v/v), combinations of Cocopeat and sawdust (3:1, v/v), and combinations of Cocopeat and compost (3:1, v/v) respectively. A beneficial effect is indicated by the colored, while a detrimental effect is represented by the color blue. The outcomes of the two-way cluster analysis also reveal that the rice straw 100% (A-2), Compost 100% (A-4), and Cocopeat 100% (A-1) treatments positively influenced most of the parameters examined. Conversely, the Sawdust 100% (A-3), Coco 75%: Rice straw 25% (C-1), and Coco 50%: Compost 50% (B-3) treatments exhibited more adverse effects on all the assessed measurements (Fig. 6).

**Fig. 7:**
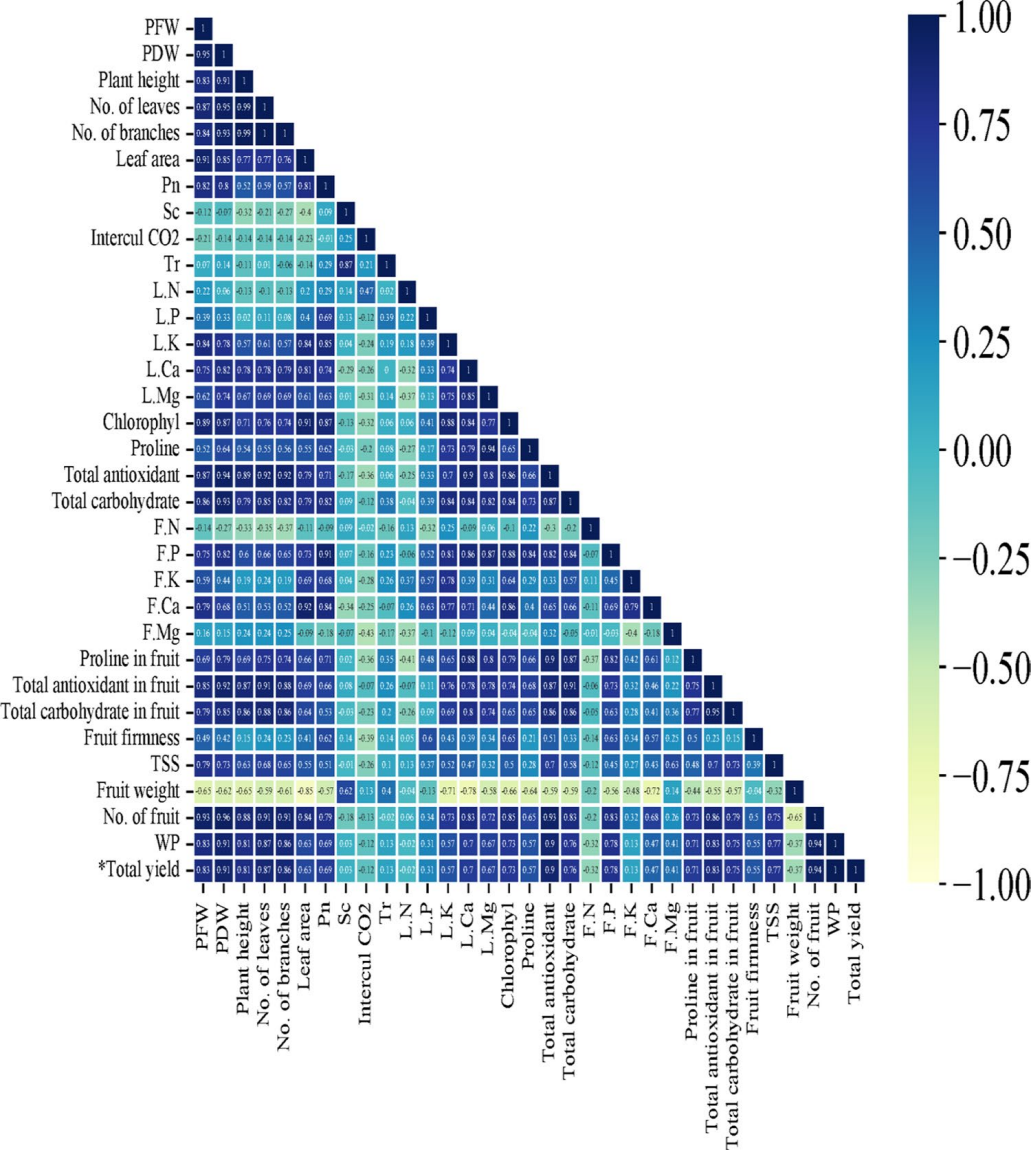
Pearson’s correlation matrix between vegetative growth parameters, quality parameters, and irrigation parameters of cucumber plants treated with different growth media.

Likewise, as shown in Fig. 7, Pearson’s correlation analysis was employed to determine the positive and negative relationships among the parameters being studied. The findings from the Pearson’s correlation analysis revealed a strong positive association between irrigation water productivity (IWP) and the following parameters: total yield, dry weight, total antioxidants in leaves, number of leaves, number of branches, fresh weight, total antioxidants in fruits, phosphorus in fruit, total soluble solids (TSS), total carbohydrates in leaves, total carbohydrates in fruits, and chlorophyll. In contrast, there exists a negative correlation between IWP and nitrogen content, recorded as -0.32.

###  Feasibility study

The economic indicators largely depend on cucumber productivity (Table 8). The production costs assess the quantity of investments made, as their values range from 7962.92 US$ ha^−1^ by Compost to 22950.56 US$ ha^−1^ by Cocopeat. Differences in the value of the production costs are evident following the different types of growth media. The highest gross revenue obtained was 28902.76 US$ ha^−1^ by Rice straw growing media, corresponding to the highest productivity achieved by 100.21-ton ha^−1,^ followed by Coco 75%: Compost 25% treatment with 21351.87 US$ ha^−1^. The lowest income obtained was under Sawdust by 5142.56 US$ ha^−1^, corresponding to the lowest productivity of 17.83 tons ha^−1^ (Table 8).

The high gross profit is an indicator estimating the economic efficiency of production. Assessing economic efficiency needs a comparison of the impact with the production costs incurred to achieve it. Thus, treated rice straw appeared as the most economically effective treatment with a profitability ratio of about 170%, followed by Compost by 119%, Coco 50%: Compost 50% with 26%, and finally Coco 50%: Compost 50% by 11%. However, the remaining treatments are found to be economically inefficient.

**Table 8:** Profitability ratios of alternative growth media treatments.

Growth media	Average yield (Mg ha^− 1^)	Production costs (US$ ha^− 1^)	Gross revenue (US$ ha^− 1^)	Gross profit (US$ ha^− 1^)	Profitability Ratio (%)
Cocopeat (control)	64.91	22950.56	18721.47	-4229.09	-18.43
Rice straw	100.21	10702.93	28902.76	18199.84	170.04
Sawdust	17.83	8120.52	5142.56	-2977.96	-36.67
Compost	60.56	7962.92	17466.83	9503.91	119.35
Coco 50%: Rice straw 50%	45.91	16826.74	13241.45	-3585.29	-21.31
Coco 75%: Rice straw 25%	54.33	19888.75	15669.96	-4218.79	-21.21
Coco 50% : Sawdust 50%	43.66	15535.64	12592.50	-2943.14	-18.94
Coco 75% : Sawdust 25%	45.41	19243.10	13097.24	-6145.86	-31.94
Coco 50% : Compost 50%	67.47	15456.74	19459.83	4003.09	25.90
Coco 75% : Compost 25%	74.03	19203.75	21351.87	2148.13	11.19

The decision criteria, NPV and B/C ratios are calculated for the economically profitable treatments as investment alternatives, with a time period of 20 years and discount rate of 12%. The findings presented in Table 9 confirm the fact that rice straw was the most economically feasible treatment in terms of NPV 135.94 thousand US$ ha^−1^ and B/C ratio of 2.70%, followed by compost with NPV 70.99 thousand US$ ha-1 and B/C of 2.19%, Coco 50%: Compost 50% by 29.90 thousand US$ ha^-1^ NRV and 1.26% B/C, and Coco 75%: Compost 25% by NPV 16.05 thousand US$ ha^−1^ and B/C equal to 1.11%.

**Table 9:** Ranking of the most profitable treatments using the financial criteria.

Growth media	NPV (Thousand US$ ha^− 1^)	B/C (%)
Rice straw	135.94	2.70
Compost	70.99	2.19
Coco 50% : Compost 50%	29.90	1.26
Coco 75% : Compost 25%	16.05	1.11
Sensitivity Analysis Scenario 1: A 25% rise in costs		
Rice straw	115.96	2.16
Compost	56.12	1.75
Coco 50% : Compost 50%	1.04	1.01
Coco 75% : Compost 25%	-19.81	0.89
Sensitivity Analysis Scenario 2: A 25% reduction in revenues		
Rice straw	81.97	2.03
Compost	38.37	1.65
Coco 50% : Compost 50%	-6.44	0.94
Coco 75% : Compost 25%	-23.83	0.83
Sensitivity Analysis Scenario 3: Scenario 1 + Scenario 2		
Rice straw	61.98	1.62
Compost	23.50	1.32
Coco 50% : Compost 50%	-35.30	0.76
Coco 75% : Compost 25%	-59.69	0.67

NPV: Net Present value.

B/C: Benefit - Cost Ratio.

The sensitivity analyses (Table 9) performed using a range of ± 25% in both costs and revenues, indicated that the reduction in revenues (Scenario 2) has a higher effect on the feasibility of the investment than the rise in production costs (Scenario 1). The combined scenarios (Scenario 3) help assess the investment’s robustness under the first and second scenarios together. In this regard, the results imply that Rice straw and compost continue to show resilience to all risks, with Rice straw being more financially viable and profitable. On the other hand, Coco 50%: Compost 50% shows resilience only under the first scenario, yet Coco 75%: Compost 25% was found to have no resilience under all prescribed scenarios.

## Discussions

Previous investigations that compared treated rice straw waste to alternative growth media in soilless culture concluded that straw waste serves as a viable substrate due to its favorable physicochemical properties, availability, and cost-effectiveness^43^. Consequently, straw waste, sawdust, and compost may represent innovative alternatives to traditional growing substrates. Thus, rice straw waste could offer a new medium to replace other growth media. The present study evaluates the feasibility of using rice straw, either independently or in conjunction with coconut fiber, as a growth medium by assessing the morphological, physiological, and productive characteristics of cucumber plants in comparison with the most prevalent substrate (coco-peat fiber) under both soilless and conventional soil conditions. To assess the viability of rice straw, sawdust, and compost, the chemical properties of the growth media were analyzed (Table 1).

The agro-physiological and biochemical characteristics, total yield, and fruit quality of cucumber plants were assessed. After two months, the growth metrics of cucumber plants revealed significant differences among the substrates utilized (Table 2). Treated rice straw significantly enhanced the growth parameters of cucumber plants, including plant height, leaf count, branch number, fresh and dry weight, and leaf area relative to the local soil substrate (Coco-peat fiber). Moreover, compost and coco-peat coupled with compost, also positively impacted plant growth parameters, albeit to a lesser extent than treated rice straw substrate when compared to the coco-peat fiber alone. These results align with prior research indicating that the application of nitrogen fertilizers in rice straw substrates markedly improves plant growth, photosynthetic efficiency, and overall crop yield^44^. The enhanced vegetative growth in cucumber plants cultivated in treated rice straw substrates may be attributed to their superior water retention ability and low carbon-to-nitrogen ratio. These factors contribute to minimizing nutrient loss, particularly nitrogen, through leaching while providing essential water and nutriewith plant demands^44–46^.

The substrate serves as the primary nutrient medium for the growth of plants in soilless systems. Abd El-Baset^47^ illustrated that varying substrate compositions influence plant growth differently due to discrepancies in water retention capacity. In our study, the moisture content measured 40% for rice straw, followed by coco-peat at 38.3%, indicative of a high capacity for water conservation and, consequently, enhanced irrigation water productivity. For both growing seasons, the rice straw medium yielded the highest irrigation water productivity, followed by a 50% mix of coco-peat and rice straw, whereas the sawdust treatment resulted in the lowest irrigation water productivity across both growing seasons. Significant variations in yield and irrigation water productivity were observed among all treatments (Table 5).

The fundamental activity of roots illustrates the capacity of flora to uptake water and essential nutrients, with root volume acting as a metric for plant vigor, while both dry and fresh weight assessments signify the accumulation of biomass in plants^48^. Conversely, the root-to-shoot ratio offers valuable insights into the hydric conditions of the root environment as well as the efficiency of water usage^49,50^. Qian, Hong^51^ established that a robust seedling index signifies the accumulation of dry matter, along with plant height, stem girth, and both dry and fresh weights of the plants. Collectively, these elements enhance the robustness of the seedling index. In the present investigation, media composed of rice straw yielded the most substantial measures for plant dry weight, fresh weight, height, and leaf area index (LAI), with compost following closely when compared to alternative treatments. Moreover, rice straw media markedly improved the growth parameters of cucumber, thereby promoting overall cucumber growth and achieving the highest yield of 99.87 tons per hectare, succeeded by treatments combining Coco 50%: Rice straw 50% and Coco 50%: Compost 50%, which produced 73.75 tons per hectare and 65.68 tons per hectare, respectively.

Photosynthesis is a pivotal mechanism for synthesizing organic compounds vital for plant growth, serving as the cornerstone for plant development^52^. The efficacy of photosynthesis is closely associated with chlorophyll content, where the concentration of chlorophyll in foliage directly influences a plant’s capacity to harness and convert photosynthesis^53^. A discernible linear correlation exists between the photosynthetic rate and stomatal conductance; as the photosynthetic rate ascends, stomatal conductance likewise increases, whilst the obstruction of photosynthesis results in diminished stomatal conductance, consequently reducing CO_2_ influx into the leaves^54^. In this investigation, the rice straw treatment demonstrated the highest photosynthetic rates, recording values of 23.34 µmol m^–2^ s^–1^ and 22.14 µmol m^–2^ s^–1^, respectively (Fig. 2A). Furthermore, stomatal conductance, transpiration rates, and intercellular CO_2_ concentrations reached their zenith under the rice straw growth medium (Fig. 2B, C, D). These results imply that the capacity for light energy conversion and the photosynthetic activity of leaf tissues were maximized when rice straw media was incorporated at a concentration of 100%.

Additionally, parameters associated with photosynthesis are regarded as critical determinants of plant performance. The findings of this study revealed enhancements in leaf chlorophyll content (SPAD value), photosynthetic rates, stomatal conductance, and intercellular CO_2_ concentration in cucumber plants cultivated in treated rice straw substrates, followed by those grown in compost substrates, whether used independently or in conjunction with Cocopeat. Similarly, numerous researchers have documented the beneficial effects of organic substrates on chlorophyll content and photosynthetic metrics^44,55^. An elevated photosynthetic rate may be linked to an increase in chlorophyll pigments, leaf area, and leaf count^56,57^, as corroborated by Pearson’s correlation analysis in this study (Fig. 7).

Numerous investigations have substantiated that the diminished bulk density and elevated porosity of rice straw and compost facilitate root penetration into the substrate, thereby allowing greater volumetric and spatial occupancy and enhancing nutrient absorption and water uptake. This improvement contributes to the efficiency of photosynthesis and overall growth performance in plants^44,55^. Furthermore, enhanced photosynthetic efficiency in foliar tissues results in increased metabolite accumulation in both leaves and fruits under both optimal and stressful conditions^55,58^.

In the current research, elevated levels of carbohydrates and total antioxidants were detected in substrates comprising rice straw and compost, utilized either independently or in conjunction with coco-peat. These findings may be attributable to the enhancement of photosynthesis efficiency^59^. A study by Iloki Assanga, Lewis Lujan^60,61^ found that when Noni (*Morinda citrifolia* L.) was grown from May to June, it exhibited weaker antioxidant activity despite a higher TPC value than when it was grown in November. This also concurred with the proportional increase in the antioxidant activity of some fruit, such as red Raspberries and Noni, during maturation^60^. In the present study, in both growing seasons, rice straw had the highest antioxidant content in fruit by 3.19 µg g^− 1^ FW and 3.22 µg g^− 1^ FW respectively, followed by Coco 50%: Compost 50% with 2.89 µg g^− 1^ FW in the 2nd growing season. On the other hand, the lowest fruit antioxidant content was observed in the 1st growing season under sawdust treatment at 2.46 µg g^− 1^ FW, and in the second growing season with Cocopeat treatment with 2.57 µg g^− 1^ FW.

Additionally, Pearson’s correlation analysis established a positive relationship between the rate of photosynthesis and the total carbohydrate and total antioxidant levels in the leaves of cucumber plants (Fig. 7). Multiple researchers have corroborated that an improved nutritional profile of plants, particularly concerning nitrogen, potassium, and phosphorus content, alongside an increased leaf photosynthesis rate, fosters the synthesis and accumulation of various metabolites, including carbohydrates and total antioxidant activity, across a range of crops^62–64^. Moreover, the concentrations of phytohormones such as IAA and GA3 were found to be elevated in plants cultivated in rice straw and compost, whether independently or in combination with coco-peat fibers (Figs. 4 A, B).

Phytohormones play a pivotal role in regulating plant growth and development. The influence of different substrate types on the production of IAA, GA3, and ABA remains ambiguous. Nonetheless, it is recognized that environmental factors—such as light, temperature, water availability, nutrient accessibility, salinity, and soil or substrate acidity—affect plant hormone levels^63,65,66^. Furthermore, numerous studies have shown that environmental stresses, including drought, nutrient scarcity, salinity, and soil or substrate acidity, significantly diminish IAA and GA3 levels while increasing ABA concentrations in various crops^63,67^. The production of plant hormones, such as IAA and GA3, is influenced by the physicochemical characteristics of growth substrates, including their water retention capacity, nutrient composition, pH, electrical conductivity, temperature, and microbial populations^67^.

The observed increases in IAA and GA3 in plants cultivated in treated rice straw and compost, whether utilized alone or in conjunction with coco-peat fibers, may be linked to their low bulk density and high capacity for water and nutrient retention^68,69^. Conversely, the elevated ABA levels (Fig. 4C) detected in the sawdust medium, compared to all other treatments, may be associated with its reduced air porosity and cation exchange capacity. These properties hinder root proliferation within the medium and impose stress on the plants, leading to increased ABA production. Additionally, plants grown in treated rice straw and compost substrates, whether alone or in combination with coco-peat fibers, exhibited a greater number of fruits, increased fruit weight, and higher total yield per plant compared to those grown in alternative substrates.

The optimal conditions concerning bulk density and porosity within the treated rice straw substrate enabled plant roots to easily penetrate the substrate, thereby facilitating the provision of water, air, and essential nutrients required for plant development, which subsequently resulted in enhanced growth and yield quantity^70^. Furthermore, the quality of cucumber fruits, encompassing total carbohydrates, total soluble solids (TSS), and total antioxidants, was positively influenced in plants cultivated with the specified substrates (treated rice straw and compost), attributable to improvements in chlorophyll content and the rate of photosynthesis^71,72^.

Bacteria and fungi generally comprise > 90% of the total soil microbial biomass, and they are responsible for the majority of soil organic matter decomposition. The ratio of fungal: bacterial biomass is particularly sensitive to soil disturbance, with lower ratios associated with increased intensity of cultivation^73^, and increased N fertilization inputs^74^. Substrate quality also alters fungal-bacterial ratios, with low quality substrates (high C/N) favoring fungi and high quality (low C/N) substrates favoring bacteria^73,75^. In soil, fungi, although numerically much less abundant than bacteria, can account for twice the weight of bacteria and actinomycetes combined^76^. In our study the C/N ration was low ranged from 20 to 30% for all substrates except for sawdust it was higher than 40% where, as represented the low C/N ratio is favorable for bacteria while the high C/N ration is favorable for fungi which demonstrated in higher nutrients and related to the carbon to nitrogen ratio is low in rice straw, followed by compost, this encourages the activity of bacteria that decompose organic matter and increase the nutrient levels in the agricultural environment, which results in increased productivity and improved vegetative and quality characteristics of cucumbers.

The negative findings of sawdust are in same line with the outcomes of Savidov, Hutchings^77^ which made it clear that fresh wood waste is rarely used as a stand-alone growth medium, although it may serve as a rooting medium for cuttings. Usually it forms a constituent (normally less than 50%) in mixtures. Sawdust has been the standard growing medium for the greenhouse industry because of its low cost and relatively high plant/fruit productivity. However, sawdust is prone to gradual decomposition, which leads to unfavorable substrate physical properties, converting it from a “dry” to “wet” substrate with a higher volume of retained water and a deficiency of available oxygen. Despite perlite’s higher stability, there was no improvement in productivity found for bell pepper or long English cucumber when compared to sawdust^77^. According to a report by Dorais, Menard^78,79^ the plant biomass, chlorophyll fluorescence and leaf area of tomato, cucumber and sweet pepper were negatively affected when grown in sawdust substrates of ten different tree species of the Canadian forest. Particularly, *Thuja* sp. based substrates substantially reduced seedling growth, leaf area, and plant biomass of tomato, cucumber and pepper vegetables because of the presence of phytotoxins such as terpenes, manganese, phenols and heavy metals in this sawdust substrate.

The correlation analysis indicated a positive relationship between the rate of photosynthesis and TSS, carbohydrates, and total antioxidants (Fig. 7). These results align with findings from various researchers who have validated those enhancements in nutrient uptake, water absorption, and photosynthetic efficiency lead to an increase in bioactive compounds across various crops^58,64^. The elevated accumulation of nutrients in both fruits and foliage of plants cultivated in treated rice straw substrates may be associated with the chemical characteristics of rice straw, particularly pH, electrical conductivity (ECe), nutrient content (nitrogen and phosphorus), and carbon-to-nitrogen (C/N) ratio (Table 1). Additionally, the physical attributes of this substrate, such as high porosity and low bulk density, facilitated the unobstructed growth of plant roots and supported the absorption of nutrients and water^30,80–82^.

Although there are many non-quantitative and non-economic criteria for making decisions, the significance of the applied analytical techniques is to improve the decision-making process, not to substitute for judgment^42^. The investment analysis methods discussed are methods to analyze economic profitability. They are meant to find an answer to the question, what is the most profitable growth media treatment? The starting point is to compare costs with benefits to determine which among the alternative treatment options have an acceptable return (Table 8). As a result, efficient ranked by profitability ratio indicator as follows: Rice straw, Compost, Coco 50%: Compost 50%, and Coco 75%: Compost 25%.

To further determine the attractiveness of the proposed investments to farmers and to the society as a whole, the analysis was projected over the useful life (20 years). The analysis concentrated on two discounted measures convenient for application in agricultural projects: net present value (NPV) and benefit-cost (B/C) ratio. Accordingly, the treatments were ranked in the same order as previously indicated by the profitability ratio indicator. Reworking the analysis to test what happens to the earning capacity of the chosen alternatives if events are different from what was expected in the planning stage, rice straw treatment is ranked first, followed by Compost treatment, in terms of resilience to all risks considered (Table 9). This investment usually has benefits beyond what is financial or economic, being socially beneficial in terms of income distribution and employment.

## Conclusions

The substrate commonly employed in soilless agriculture is primarily peat; however, due to its non-renewable nature and high costs, it is imperative to explore alternative substrates that can partially replace peat. Among these innovative options, rice straw emerges as a viable growing medium, recognized for its availability and cost-effectiveness, thereby addressing this necessity. This research investigated the physical and chemical properties of various single and composite substrates, including rice straw, Cocopeat, sawdust, and compost, and their impact on plant growth, irrigation water efficiency, photosynthetic activity, fruit quality, and carbohydrate metabolism in cucumber plants.

Findings indicated that rice straw improved substrate aeration and positively affected irrigation water productivity, growth parameters, root development, chlorophyll levels, photosynthetic traits, and nutrient content in cucumber fruits. Moreover, rice straw significantly increased the concentrations of total soluble solids, firmness, Proline, and antioxidants in the fruits.

The constraints we encountered were farmers’ lack of conviction in the feasibility of using rice straw as a growing medium in greenhouses. There is a widespread belief, not only among local farmers but also among agricultural engineers, that local products are ineffective and that imported products are less effective. Furthermore, with the rise in fertilizer prices, farmers have begun to use agricultural waste and animal waste as organic fertilizers without treating them. This eliminates harmful microbes and controls the carbon-to-nitrogen ratio, which harms agricultural soil and crops and increases emissions from the soil.

In summary, rice straw proved to be an effective local growth medium for cucumber cultivation due to its outstanding physiochemical properties. Additionally, substituting Cocopeat with rice straw not only reduces the costs of soilless cultivation but also offers beneficial economic and environmental impacts. Future research on rice straw substrates should prioritize assessing the stability of this medium across multiple cultivation cycles, which will enhance confidence in the benefits associated with such growth media.

## Data Availability

All data included in the research will be made available upon request, while the data from previous studies and research was obtained through the Cairo University platform, which provides research information on a regular basis. If someone wants to request the data from this study, kindly contact Prof. Dr. Mohamed E. Abuarab (mohamed.aboarab@agr.cuedu.eg).
