# Correction: Enhancing greenhouse cucumber production and quality utilizing organic residues as potting media through various cultivation modes and assessing application economics

**DOI:** 10.1038/s41598-025-30364-z

**Published:** 2025-12-03

**Authors:** Eman A. Gab Allah, Mohamed M. Shahein, Mohamed E. Abuarab, Victor Shaker, Emad A. Abdeldaym

**Affiliations:** 1 Department of Vegetable, Faculty of Agriculture, Cairo University, Giza, 12613 Egypt; 2 Agricultural Engineering Department, Faculty of Agriculture, Cairo University, Giza, 12613 Egypt; 3 Department of Agricultural Economics, Faculty of Agriculture, Cairo University, Giza, 12613 Egypt

Correction to: *Scientific Reports* 10.1038/s41598-025-05242-3, published online 20 June 2025

The original version of this Article contained an error in the spelling of the author Victor Shaker, which was incorrectly given as Vector Shaker.

The original Article has been corrected.

